# *Ruminococcus gnavus*: friend or foe for human health

**DOI:** 10.1093/femsre/fuad014

**Published:** 2023-04-04

**Authors:** Emmanuelle H Crost, Erika Coletto, Andrew Bell, Nathalie Juge

**Affiliations:** Quadram Institute Bioscience, Rosalind Franklin Road, Colney, Norwich NR4 7UQ, United Kingdom; Quadram Institute Bioscience, Rosalind Franklin Road, Colney, Norwich NR4 7UQ, United Kingdom; Quadram Institute Bioscience, Rosalind Franklin Road, Colney, Norwich NR4 7UQ, United Kingdom; Quadram Institute Bioscience, Rosalind Franklin Road, Colney, Norwich NR4 7UQ, United Kingdom

**Keywords:** *Ruminococcus gnavus*, mucus, gut adaptation, carbohydrate metabolism, metabolites, intestinal and extraintestinal diseases

## Abstract

*Ruminococcus gnavus* was first identified in 1974 as a strict anaerobe in the gut of healthy individuals, and for several decades, its study has been limited to specific enzymes or bacteriocins. With the advent of metagenomics, *R. gnavus* has been associated both positively and negatively with an increasing number of intestinal and extraintestinal diseases from inflammatory bowel diseases to neurological disorders. This prompted renewed interest in understanding the adaptation mechanisms of *R. gnavus* to the gut, and the molecular mediators affecting its association with health and disease. From ca. 250 publications citing *R. gnavus* since 1990, 94% were published in the last 10 years. In this review, we describe the biological characterization of *R. gnavus*, its occurrence in the infant and adult gut microbiota and the factors influencing its colonization of the gastrointestinal tract; we also discuss the current state of our knowledge on its role in host health and disease. We highlight gaps in knowledge and discuss the hypothesis that differential health outcomes associated with *R. gnavus* in the gut are strain and niche specific.

## Teaser

*Ruminoccocus gnavus*, a prevalent member of the infant and adult gut microbiota has emerged a as a key biomarker of health and diseases with functionally characterized immune and metabolic properties.

## Introduction

**Figure 1.**
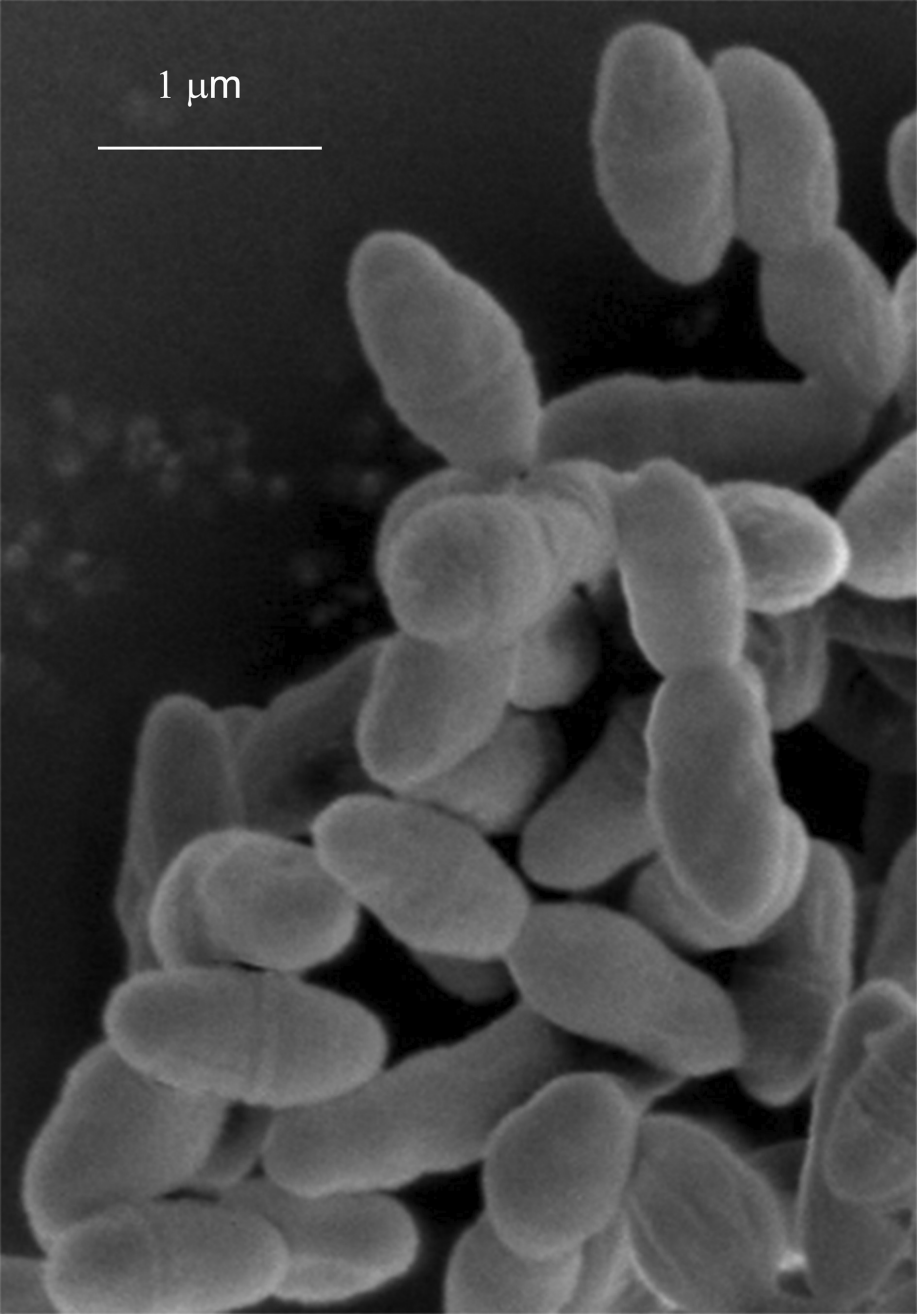
Scanning electron microscopy image of *R. gnavus* ATCC 35913 anaerobically grown in rich medium to stationary phase (courtesy of Catherine Booth).

*Ruminococcus gnavus* is a Gram-positive anaerobic bacterium belonging to the phylum Firmicutes (recently renamed Bacillota) (Ludwig et al. 2009). *R. gnavus* was first isolated from faeces and contents of the gastrointestinal (GI) tract of humans in 1974 (Moore and Holdeman 1974). The type strain [ATCC 29149 ( = VPI C7-9)] was then characterized in 1976 when it was described as obligate anaerobic, nonspore-forming, nonmotile, or motile cocci with 1–3 flagella that occurred in chains or pairs (Moore et al. 1976) (see Fig. 1)*. R. gnavus* was originally placed in the genus *Ruminococcus* because of its enhanced growth on fermentable carbohydrates, producing acetate, and formate but not butyrate as fermentation products; and because the G + C content of its genomic DNA (43 mol%) was within the range (40–45 mol%) reported previously for species in the genus *Ruminococcus* (Moore et al. 1976). The Latin adjective gna’vus (na’vus) meaning busy refers to the active fermentation capability of this species. With advances in molecular profiling, members of the genus *Ruminococcus* were later divided into two families based on 16S rRNA sequencing: Ruminococcaceae for *Ruminococcus* species belonging to *Clostridium* cluster IV, and Lachnospiraceae for species belonging to *Clostridium* cluster XIVa (Liu et al. 2008). According to this classification, *R. gnavus* is part of the Lachnospiraceae family. All members of the Lachnospiraceae are strict anaerobes but their spore-forming status varies (Dworkin et al. 2006). Historically, *R. gnavus* was reported as a nonspore former (Liu et al. 2008). However, recent work has found that many previous nonspore-forming species, including *R. gnavus*, could form spores under specific culture conditions that favour sporulation (Browne et al. 2016). In addition, while *R. gnavus* ATCC 29149 is unable to grow in the presence of oxygen, it did demonstrate some tolerance to atmospheric oxygen with 10%–15% of cells remaining viable after exposure to oxygen for 1 hour, falling to around 0.05%–0.1% after 3 hours (Hall et al. 2017). Despite retaining its genus name for study purposes, as a species, *R. gnavus* does not technically belong to the genus *Ruminococcus* (a mistake commonly encountered in metagenomics studies); for a while it was classified as belonging to the genus *Blautia* (Lawson and Finegold 2015) but has recently been reclassified as belonging to the genus *Mediterraneibacter* (Togo et al. 2018a,b).

## Microbial ecology of *R. gnavus* in the human gut

### *R. gnavus* in early life and influence of nutrition

*R. gnavus* is a prevalent member of the ‘normal’ human gut microbiota and several lines of evidence show that *R. gnavus* is prevalent from early life. During the first year of life, factors such as mode of delivery, diet, and genetics all influence microbial colonization (Rodriguez et al. 2015). In a study focusing on Lachnospiraceae in amer samples from 25 babies aged between 1 and 24 months, *R. gnavus* was identified as being prevalent using denaturing gradient gel electrophoresis (DGGE) and quantitative PCR (qPCR); specifically, *R. gnavus* was recorded in 22 of 25 (88%) samples (Sagheddu et al. 2016). This study, although limited in size, suggested that *R. gnavus* was a predominant species in the infant gut occurring at levels as high as *Bifidobacterium* spp.; and that this was not dependent on the type of delivery or feeding regime (Sagheddu et al. 2016). However, other studies using 16S rRNA gene sequencing analysis to investigate the role of breast feeding or different infant milk formulas on the infant faecal microbiota, highlighted associations between *R. gnavus* and diet. For example, in a comparative study of 90 Australian 2-month old infants (30 in each group), the presence of Lachnospiraceae was restricted to a single species, *R. gnavus*, in babies fed breast milk or goats’ milk compared with a more diverse microbial profile in babies fed cows’ milk (Tannock et al. 2013). In another proof-of-principle randomized controlled trial (RCT), the faecal microbiota of infants fed cows’ milk formula (CMF) containing lactose were compared with those fed an isocaloric extensive protein hydrolysate formula (EHF) used for infants who are intolerant to cows’ milk proteins. *R. gnavus* exhibited the greatest increase in abundance over time in the EHF group and was the most prominent signature distinguishing the microbiome of EHF – from CMF-fed infants (Mennella et al. 2022).

These data suggest that *R. gnavus* could play a role in priming the gut microbiota in association with normative weight gain velocity and weight status in infants (Mennella et al. 2022). This is supported by an earlier study revealing that *R. gnavus* was one of the 24 ‘age-discriminatory’ taxa whose changes in relative abundance over time defined normal ‘maturation’ of the microbiota of infants and children in Bangladesh (Subramanian et al. 2014). Many mechanisms have been proposed to mediate these effects including driving amino acids away from oxidation in favour of protein synthesis and lean mass formation (Blanton et al. 2016). A recent study focusing on infant gut microbiota during pregnancy and at delivery (cord blood) showed that *R. gnavus* was positively associated with prenatal plasma 25-hydroxyvitamin D (25[OH]D) but negatively associated with cord 25[OH]D (Kassem et al. 2020).

Several lines of evidence suggest that *R. gnavus* increases in relative abundance post weaning while others such as *Bifidobacterium* species are known to decrease in relative abundance (Yatsunenko et al. 2012). An early study investigating the impact of weaning on faecal microbiota composition of 605 infants from five European countries showed a significant increase in the proportion of the *Clostridium coccoides* group that *R. gnavus* belongs to after the first introduction of solid foods (Fallani et al. 2011). Short-chain fatty acids (SCFAs) are also indicative of changes in gut microbiota composition with data showing changes in butyrate production during weaning. Analysis of the faecal microbiota in a cohort of 28 Nigerian infants within the first year of life showed that breast-fed infants had a predominance of *R. gnavus, Collinsella*, and *Sutterella* species (Oyedemi et al. 2022); in the majority of infants butyrate was first detected when weaning started, at between 4 and 6 months old, while acetate and lactate remained high following the introduction of solid foods (Oyedemi et al. 2022). Analysis of faecal samples from infants at birth, 3-, 6-, and 12-months of age in the general population-based PreventADALL cohort found that low levels of butyrate at 12-months old correlated with *R. gnavus* abundance, in line with its known fermentation capacity (see section on the 'Molecular mediators underpinning the effect of *R. gnavus* on health and disease'); and that butyrate levels during the transition from an infant- to an adult-like gut microbiota correlated with bacterial networks associated with *Eubacterium rectale* and *R. gnavus* (Nilsen et al. 2020). Further, daily supplementation with a mixture of three strains of *Bifidobacterium* species, following antibiotic therapy cycles, to a 4-month-old baby with propionic acidaemia, resulted in an increase in *R. gnavus* abundance in the infant’s stools (Bordugo et al. 2021). Taken together, these studies support the role of *R. gnavus* in early colonization of the gut. The capacity of some *R. gnavus* strains to metabolize human milk oligosaccharides (HMOs) and mucins, which share structural glycan similarity (see section on the 'Adaptation mechanisms of *R. gnavus* to the gut'), supports the hypothesis that this species plays an important role in colonization of other bacteria during ageing (O’Toole and Claesson 2010).

### *R. gnavus* in adulthood and influence of nutrition

*R. gnavus* persists throughout adulthood; based on metagenomic sequencing of faecal samples from healthy North American and European adults, it is one of the 57 species present in ≥90% of individuals at a median abundance of around 0.1% (Qin et al. 2010, Kraal et al. 2014). More recently, *R. gnavus* was found in 39/60 (65%) of publicly available metagenomes of gut microbiota from healthy adults from China, Ethiopia, Spain, USA, and Sweden with a mean abundance of 0.3% (Candeliere et al. 2022). As an aside, *R. gnavus* was found to be most responsive to circadian rhythm disruption in adults (Mortas et al. 2022).

In the elderly, altered environmental and physiological factors associated with ageing may favour particular bacterial species in the gut. A study investigating how biological or chronological age relate to taxonomic differences in the gut microbiota identified a frailty-associated coabundance of *Eggerthella* (100% *E. lenta*) and *Ruminococcus* genera (99% *R. gnavus*) (Maffei et al. 2017). Microbial profiling of the gut microbiota from 90 to 99-years-old and the 100+-years-old age groups showed more diversity, robustness, and richness compared with the 65–70-years-old age group and a clear separation between the 65–70-years-old and 100+-years-old age groups. At the species level, *Bacteroides fragilis, Parabacteroides merdae, R. gnavus, Coprococcus*, and *Clostridium perfringens* increased in abundance, while *Bacteroides vulgatus, Ruminococcus* sp.5139BFAA, and *Clostridium* sp.AT5 decreased in abundance in the 90–99-years-old age group (Wang et al. 2019).

As with infants, diet is the main modulator of the gut microbiota in adults. In the study titled ‘New dietary strategies addressing the specific needs of the elderly population for healthy aging in Europe’ (NU-AGE), which investigated associations between diet, gut microbiota, and cognition, results showed that *R. gnavus* and *Collinsella* spp. were associated with animal product-rich diets and were referred to as ‘pro-inflammatory species’ (van Soest et al. 2020). Long-term associations between diet quality and the gut microbiome were determined using 16S rRNA gene sequencing of faecal samples from a multiethnic cohort study (5936 participants); the presence of *R. gnavus* was inversely associated with the healthy eating index (HEI) (Ma et al. 2022). Folate is naturally present in a wide variety of foods and folic acid plays an important role in health as it affects the growth and regulation of cell functions and low levels are a risk factor for IBD pathology (Ratajczak et al. 2021). *R. gnavus* was strongly correlated with the increase in folic acid observed in elderly people susceptible to neurocognitive disorders (Han et al. 2022b).

Few dietary intervention studies have evaluated the impact of specific foods or food components on *R. gnavus*. For example, a randomized cross-over intervention trial of 23 healthy individuals showed that adding high-amylose maize to a high red meat diet lowered the proportions of *R. gnavus, Ruminococcus torques* and *Escherichia coli* (Le Leu et al. 2015) while supplementation with (poly)phenol-dense red raspberries decreased *R. gnavus* in adults with prediabetes and insulin resistance (IR; see section 2.4) (Zhang et al. 2022), suggesting that dietary interventions targeting *R. gnavus* may be used as a strategy to promote health.

### Adaptation mechanisms of *R. gnavus* to the gut: microbial colonization factors

The ability of symbiotic bacteria to colonize the gut is mediated by several mechanisms including direct killing, competition for nutrients, and adhesion. To colonize and persist in the gut, *R. gnavus* has evolved strain-specific strategies to adapt to this specific milieu by producing bacteriocins to kill competitors, glycoside hydrolases (GH) active on a range of dietary or host carbohydrates, and adhesins to mucus, as decribed below.

#### Bacteriocins

Bacteriocins are ribosomally synthesized antimicrobial peptides secreted by bacteria to kill other bacteria and therefore inhibit the colonization and growth of other species. First evidence of bacteriocin production in *R. gnavus* came from studies on *R. gnavus* E1, a strain isolated for its anti-*C. perfringens* activity from the gut microbiota of a healthy adult man (Ramare et al. 1993). This antimicrobial activity was later associated with the production of bacteriocins called ruminococcins. Ruminococcin A (RumA), a bacteriocin belonging to the lantibiotic family was the first of the E1 antimicrobial peptides to be characterized (Dabard et al. 2001). RumA showed activity against several pathogenic *Clostridium* spp. (including some strains of *C. difficile*) and also species phylogenetically related to *R. gnavus* (including the type strain ATCC 29149) (Ramare et al. 1993, Dabard et al. 2001). *RumA* genes are widely represented amongst phylogeneticaly related bacteria in the human gut microbiota (Marcille et al. 2002). In addition, genetic transfer of *rumA* genes from *R. gnavus* E1 to a strain of *Dorea longicatena* was observed in the digestive tract of gnotobiotic mice (Crost et al. 2010), supporting the presence of the *rumA* biosynthetic gene cluster on a mobile genetic element (Gomez et al. 2002b). Structural chacterization of recombinant RumA confirmed the presence of two thioether bridges and revealed the presence of a third methyllanthionine bridge (Ongey et al. 2018). Expression of *rumA* is under the control of a two-component system activated by trypsin (Gomez et al. 2002a) but low in the gut of *R. gnavus* E1 monoxenic rats (Crost et al. 2011), suggesting that this *R. gnavus* strain encodes another anti-*C. perfringens* substance.

Another ruminoccin gene, *rumB*, has been identified but is not expressed by *R. gnavus* E1 due to an insertion in the sequence (Gomez et al. 2002b). Several bacteriocins have subsequently been purified from the caecal content of *R. gnavus* E1 monoxenic rats (Crost et al. 2011) and found to correspond to predicted peptides encoded by five genes, *rumC1*-*5* (Crost et al. 2011, Pujol et al. 2011). The *rumC* biosynthetic gene cluster is in the vicinity of the *rumA* biosynthetic gene cluster and also thought to be on a mobile genetic element. All *rumC1*-*5* genes were transcribed in the gut of monoxenic rats but not *in vitro*, even when grown in the presence of trypsin (Pujol et al. 2011). RumC bacteriocins were further purified and characterized as sactipeptides with four sulfur-to-a-carbon thioether bridges (Balty et al. 2019, Chiumento et al. 2019).

Most recent studies have focussed on the bacteriocin RumC1 isolated from *R. gnavus* E1. Due to its thioether bridges, this peptide is highly stable at low and high pH, high temperature, and high salt concentrations, which are beneficial features for pharmaceutical/industrial application. Recombinant RumC1 was found to be active against *C. perfringens, C. difficile, C. botulinum, Listeria monocytogenes, B. cereus*, vancomycin-resistant *Enterococcus faecalis*, nisin-resistant *B. subtilis* and methicillin-resistant *Staphylococcus aureus*, although results varied amongst studies (Balty et al. 2019, Chiumento et al. 2019, Roblin et al. 2020). It has been suggested that RumC1 does not act as a pore-forming bacteriocin but might inhibit the ATP synthesis pathway (Chiumento et al. 2019, Roblin et al. 2020). RumC1 showed no toxicity to intestinal, colonic, or gastric cell lines or to human intestinal explants (Chiumento et al. 2019, Roblin et al. 2020) and was even reported to have beneficial effects on host cells (Roblin et al. 2021). Interestingly, RumC1 performed as well as vancomycin in relation to survival, overall health and blood markers, in a mouse model intraperinoteally infected with a clinical isolate of *C. perfringens* (Roblin et al. 2021). RumC1 was able to kill *C. perfringens* in a complex microbial environment although it did also affect other members of the bacterial community, mostly by reducing the abundance of strains in the *Clostridium* cluster XIVa. The changes in gut microbiota composition observed led to an increase in butyrate and acetate and a decrease in propionate and ammonia, suggesting that *R. gnavus* E1 may have a beneficial impact on gut homeostasis via RumC1. Further work is warranted to determine the biological drivers for the strain-specific occurrence of bacteriocins amongs *R. gnavus* strains.

#### Host and dietary carbohydrate metabolism

**Figure 2.**
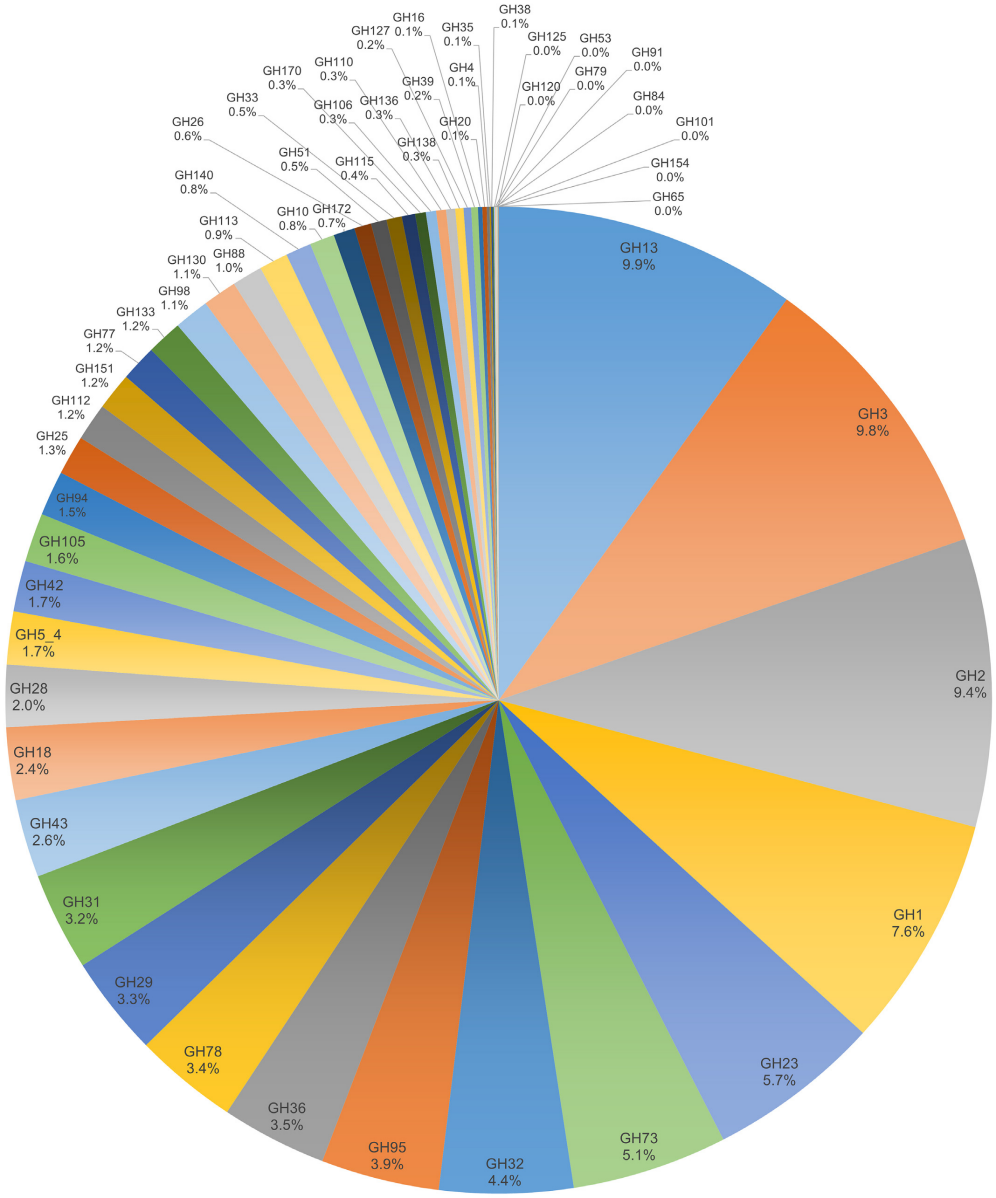
Distribution of GHs across *R. gnavus* strains. A total of 7741 putative GHs has been identified by analysing 92 genomes of *R. gnavus* strains. These GHs are spread across 56 GH families. Some GH families are not widely spread amongst *R. gnavus* strains (i.e. GH35, GH38, GH53, GH65, GH79, GH84, GH91, GH101, GH120, GH125, and GH154) while other families are present in all the strains (i.e. GH1, GH2, GH3, GH13, GH18, GH25, GH29, GH31, GH32, GH36, GH73, GH77, GH78, GH95, GH112, and GH151). More than half of the sequences (4021/7741) belongs to seven GH families: GH1, GH2, GH3, GH13, GH23, GH32, and GH73. Combined, these families cover enzymes with a wide range of activities including (but not exclusively) α-amylase, isoamylase, pullulanase, β-glucosidase, β-galactosidase, β-mannosidase, β-glucuronidase, β-xylosidase, lysozyme, peptidoglycan hydrolase, invertase, inulinase, and endo-levanase.

Dietary and host carbohydrates shape the gut microbiota by providing a major source of nutrient for microbes inhabiting the gut. The ability of *R. gnavus* to use complex carbohydrates has been investigated in relation to host glycans such as mucin glycans and HMOs (Crost et al. 2013, 2016, Wu et al. 2021b), as well as dietary carbohydrates such as oligosaccharides (Bruel et al. 2011) and resistant starch (Crost et al. 2018). To deconstruct and metabolize complex glycans, *R. gnavus* strains produce a range of carbohydrate-active enzymes (CAZymes) including GHs, glycosyltransferases (GT), polysaccharide lyases (PL), and carbohydrate esterases (CE) (www.cazy.org). Bioinformatics analyses of all 92 *R. gnavus* genomes currently available showed that 56/172 GH, 9/115 GT, 2/42 PL, and 9/20 CE families were represented. The total number of CAZymes varies between *R. gnavus* strains (i.e. from 60 to 116 GH families per strain) (see Fig. 2). Although this is higher than the average number in Firmicutes (39.6 mean number of GH and PL genes per genome), this number is inferior to the proportion of GHs found in Bacteroidetes species (137.1 mean number of GH and PL genes per genome), which can switch from host to dietary carbohydrate sources (El Kaoutari et al. 2013, Garron and Henrissat 2019). This also reflects differences in the strategies used for complex carbohydrate utilization by Bacteroidetes and Firmicutes. In Bacteroidetes, CAZymes are grouped within polysaccharide utilization loci (PULs) allowing disassembly of complex polysaccharides in a highly coordinated process while Firmicutes encode a lower proportional number of CAZymes that are specialized to target a few selected carbohydrates (Ndeh and Gilbert 2018, La Rosa et al. 2022).

##### Mucin glycan and HMO utilization

Mucin glycans provide a reliable source of sugars and nutrients for a range of bacterial species. Mucin O-glycans consist of N-acetylgalactosamine (GalNAc), Gal, and N-acetylglucosamine (GlcNAc) containing glycan chains usually capped by fucose (Fuc) and/or sialic acid (Neu5Ac), giving rise to blood group A, B, and H antigens and sialyl-Lewis epitopes. The first reports of *R. gnavus*’ capacity to degrade mucin glycans were from Hoskins et al. (1985), who isolated *R. gnavus* ATCC 35913 (formerly known as *Ruminococcus AB* strain VI-268) from a faecal sample of a healthy blood group B secretor. This strain was shown to partly degrade pig gastric mucins and produce blood group B-degrading α-1,3- galactosidase, blood group H-degrading α-1,2- fucosidase, α-1,4- fucosidase, α-2,3- sialidase, sialate O-acetylesterase, and glycosulfatase (Hoskins et al. 1985, Larson et al. 1988, Corfield et al. 1992). More recently, we have shown that the ability of *R. gnavus* to utilize mucin glycans and HMOs is strain specific (Crost et al. 2013, 2016). Out of the three strains evaluated experimentally, *R. gnavus* ATCC 29149 and ATCC 35913 but not *R. gnavus* E1 could grow on mucin as the sole carbon source (Crost et al. 2013, 2016). When grown on HMOs, *R. gnavus* E1 was able to use 2′-fucosyllactose (2′FL), the most abundant HMO in human breast milk, as well as 3-fucosyllactose (3FL) and *N*-acetyllactosamine (LacNAc), while ATCC 29149 could use 2′FL, 3FL, and 3′-sialyllactose (3′SL). Neither strains could use lacto-N-tetraose (LNT), lacto-N-neotetraose (LNnT), or 6′-sialyllactose (6′SL) (Crost et al. 2013). Strain differences in the ability of *R. gnavus* to grow on mucins may be related to the presence of GH33 sialidase in *R. gnavus* ATCC 29149 and ATCC 35913 but not in E1, which is supported by the growth patterns of these strains on 3′SL. Further transcriptomics analysis on mucin and HMOs revealed induction of several GH families including sialidases (GH33), β-galactosidases (GH2), fucosidases (GH95), blood group endo-β-1,4-galactosidase (GH98), and sialic acid utilization genes (Crost et al. 2013, 2016, Wu et al. 2021b). Several of these enzymes have been biochemically and structurally characterized in *R. gnavus* ATCC 29149 and/or *R. gnavus* E1 strains (Table 1) as described in detail below.

**Table 1.** Functionally characterized *R. gnavus* GHs.

GH family (activity)	Name	Gene accession number	Strain	Substrate	PDB	EC number	References
GH2 (β-glucuronidase)	UidA or GusA or *Rg*GUS	GenBank AY307023	E1	pNP-β-D-glucuronide	5Z18-9 6EC6 6JZ1-8	3.2.1.31	Beaud et al. (2005)
GH36 (α-galactosidase)	Aga1	ENA ACL13770.1	E1	Melibiose, raffinose, stachyose		3.2.1.22	Aguilera et al. (2012), Cervera-Tison et al. (2012)
GH36 (α-galactosidase)	Aga2	GenBank FO203362	E1	Melibiose, raffinose, stachyose		3.2.1.22	Cervera-Tison et al. (2012)
GH36 (α-galactosidase)	AgaSK	ENA FQ790379	E1	Melibiose, raffinose, stachyose	2YFN 2YFO	3.2.1.22	Bruel et al. (2011), Lafond et al. (2020)
GH13_18 (sucrose 6F-phosphate phosphorylase)	*Rg*SPP	ENA FQ790378	E1	Sucrose 6F-phosphate		2.4.1.329	Tauzin et al. (2019)
GH33 (intramolecular *trans*-sialidase)	*Rg*NanH	GenBank QEI30999.1	ATCC 29149	α-2,3-sialylconjugates	4 × 4A 4 × 49 4 × 6 K 4 × 47	4.2.2.15	Tailford et al. (2015)
GH98 (blood-group A endo-β-1,4-galactosidase	*Rg*GH98	GenBank QEI31357.1	ATCC 29149	BgAtetra type II	7Q20 7PMO 7Q1W	3.2.1.8	Wu et al. (2021b)
GH29 (α-L-fucosidase)	RUGNEv3_10125		E1	pNP-Fuc, 3FL, LeA, LeX	6TR3 6TR4	3.2.1.51	Wu et al. (2021a)
GH29 (α-L-fucosidase)	RUGNEv3_10180		E1	pNP-Fuc, 3FL, LeA, LeX		3.2.1.51	Wu et al. (2021a)
GH29 (α-L-fucosidase)	RUMGNA_03833	GenBank QEI30547.1	ATCC 29149	pNP-Fuc, 2′FL, 3FL, LeA		3.2.1.51	Wu et al. (2021a)
GH95 (α-L-fucosidase)	RUGNEv3_10587		E1	pNP-Fuc		3.2.1.51	Wu et al. (2021a)
GH95 (α-L-fucosidase)	RUMGNA_00842	GenBank AAYG02000007.1	ATCC 29149	pNP-Fuc, 2′FL, 3FL		3.2.1.51	Wu et al. (2021a)

*R. gnavus* ATCC 29149 encodes a GH33 enzyme (*Rg*NanH) that has a unique enzymatic activity in gut symbionts, whereby it releases 2,7-anhydro-Neu5Ac instead of Neu5Ac from glycoconjugates linked to an α2,3-terminal sialic acid residue, classifying this enzyme as an intramolecular *trans*-sialidase (Tailford et al. 2015). *Rg*NanH was shown to be active against 3′SL but not 6SL, in line with the growth data (above), but also 3′-sialyl-3-fucosyllactose, 3′-α-sialyl-*N*-acetyllactosamine (3′SLacNAc), 3′-sialyl lewis X, and 3′-sialylgalactose, suggesting a role in host glycan utilization including mucins and HMOs (Tailford et al. 2015). The gene encoding *Rg*NanH is part of a *nan* operon dedicated to 2,7-anhydro-Neu5Ac utilization which encodes an ABC transporter dedicated to the transport of 2,7-anhydro-Neu5Ac into the cells via the exquisite selectivity of the solute binding protein (*Rg*SBP) for this sialic acid form (Bell et al. 2019) and an intracellular oxidoreductase (*Rg*NanOx) converting it back to Neu5Ac before being metabolized through the Neu5Ac canonical metabolism pathway (Bell et al. 2020). Due to its terminal location and abundance in mucins, sialic acid is a highly sought-after nutrient and many bacteria have evolved strategies to use it (Juge et al. 2016). This is also supported by bioinformatics analyses showing emergence of several classes of transporters with sialic acid specificity across bacteria, underscoring the importance of developing competitive strategies to acquire host-derived sialic acid for successful colonization (Severi et al. 2021). It has been proposed that this unique sialic acid metabolism pathway among gut bacteria confers *R. gnavus* strains with a nutritional competitive advantage by releasing sialic acid in a form, 2,7-anhydro-Neu5Ac, that it can preferentially (‘selfishly’) use. The biological importance of this peculiar sialic acid metabolic pathway in *R. gnavus* was confirmed by generating a *R. gnavus* ATCC 29149 *nan* deletion mutant that exhibited impaired fitness and reduced ability to colonize the mucus layer in gnotobiotic mice (Bell et al. 2019).

*R. gnavus* ATCC 29149 also encodes a GH98 enzyme (*Rg*GH98), which is not present in the non-mucin glycan foraging E1 strain. *Rg*GH98 has substrate specificity for blood group A tetrasaccharide (BgAtetra), as determined enzymatically and by isothermal titration calorimetry and saturation transfer difference (STD) NMR (Wu et al. 2021b). The gene encoding *Rg*GH98 is part of an operon of ten genes that are overexpressed *in vitro* when *R. gnavus* ATCC 29149 is grown on mucin as the sole carbon source. This cluster is predicted to encode other CAZYmes including GH95 fucosidase and a putative GH73 endo-β-*N*-acetylglucosaminidase. Furthermore, pretreatment of mucin with *Rg*GH98 conferred *R. gnavus* E1 with the ability to grow, by enabling it to metabolize blood group A trisaccharide (BgAtri) and access the underlying mucin glycan chain, as confirmed by MALDI-ToF MS (Wu et al. 2021b). Specificity of *Rg*GH98 for BgA antigens may provide *R. gnavus* with an advantage in colonizing blood group A individuals with secretor status (i.e. indicative of the presence of blood group antigens in mucus) in the population. This activity may also influence *R. gnavus* strain acquisition in infants since the HMO profile of the mother is determined by both secretor and Lewis status based on polymorphisms in genes encoding the FUT2 and FUT3 enzymes that generate Lewis antigens, perhaps contributing to early adaptation of *R. gnavus* to the infant gut (see section on 'Microbial ecology of *R. gnavus*in the human gut').

Fucosidase activity has been investigated in both *R. gnavus* ATCC 29149 and E1 strains (Crost et al. 2013, Wu et al. 2021a). *R. gnavus* E1 harbours four GH29 and four GH95 encoding genes while two GH29 and three GH95 were present in the genome of *R. gnavus* ATCC 29149. Sequence similarity network analyses identified strain-specific fucosidases in both *R. gnavus* ATCC 29149 and E1 strains that were further characterized enzymatically against a range of defined oligosaccharides and glycoconjugates. A total of five fucosidases with activity against p-nitrophenyl-α-L-fucopyranoside (pNP-Fuc) were further characterized on a range of fucosylated ligands including 2′FL, 3FL, Lewis A, and Lewis X (Wu et al. 2021a). RUGNEv3_10587 from E1 was only active against pNP-Fuc, whereas RUGNEv3_10125 and RUGNEv3_10180 from E1 showed a preference for α1,3/4 fucosylated linkages and RUMGNA_00842 and RUMGNA_03833 from ATCC 29149 showed a preference for α1,2 linkages, suggesting a role of these fucosidases in supporting growth of *R. gnavus* ATCC 29149 and E1 on both 2′FL and 3FL (above), and also degrading HMOs in the infant gut. RUGNEv3_10125 also had the capacity to recognize α-1,3 and α-1,4 fucosylated substrates containing a terminal sialic acid modification (Wu et al. 2021a). The peripheral terminal epitopes of mucins show considerable variation with a decreasing gradient of Fuc and ABH blood group expression and an increasing gradient of sialic acid from the ileum to the colon in humans while the reverse gradient was observed in mice (Robbe et al. 2003, 2004, Larsson et al. 2009, 2013). *R. gnavus* fucosidase specificities may contribute to spatial adaptation of *R. gnavus* strains to different regions of the GI tract.

Together, these data support the capacity of *R. gnavus* strains to colonize different nutritional niches in the infant and adult human gut via HMO utilization and mucin glycan foraging. *R. gnavus*’ strategy to colonize mucus is based on its capacity to forage mucin glycan epitopes such as sialic acid, fucose, or blood group antigens (Bell and Juge 2021). Uncapping of mucin glycan chains through the combined action of GH33, GH98, GH29, and GH95 enzymes not only releases sugars for *R. gnavus* consumption but could also provide gut bacteria such as *Akkermansia muciniphila* or *Bacteroides* species further access to the underlying mucin glycan chains enabled by their extensive GH arsenal (Ndeh and Gilbert 2018, Bell and Juge 2021, Berkhout et al. 2022). Due to their foraging activity on mucin glycan epitopes, the *R. gnavus* mucin glycan degradation strategy is particularly well-adapted to mucin with short glycan chains, which are found in higher proportions in IBD patients (Larsson et al. 2011). This may be one of the factors contributing to an over-representation of *R. gnavus* in this disease, which is in marked contrast to *A. muciniphila*, which shows the reverse phenotype in IBD and is more adept at degradating fully glycosylated mucin chains (see section on the 'Association between *R. gnavus* and diseases').

##### Dietary glycan utilization

*R. gnavus* has mainly been studied for its ability to grow on host mucin. Unlike *Bacteroides* species for which PULs have been characterized for a wide range of plant cell wall polysaccharides, such as pectins, xylans, arabinogalactan proteins, xyloglucan, or mannans, as well as major plant storage carbohydrate such as starch and fructans (Ndeh and Gilbert 2018), only limited information is available on the range of plant polysaccharides that *R. gnavus* can use and on the GHs involved in their degradation (Table 1).

*R. gnavus* E1 can grow on melibiose and raffinose as the sole carbon source; α-galactosides such as melibiose, raffinose, stachyose, and galactomannans consist of galactose units α-(1,6) linked to a glucose, sucrose, raffinose, or mannopyranose backbone, respectively. They are mostly found in plant cell walls and are abundant oligosaccharides in our diet. Three enzymes with α-1,6-galactosidase activity have been functionally characterized in *R. gnavus* E1: Aga1 (Aguilera et al. 2012) and Aga2 (Cervera-Tison et al. 2012), which share approximately 45% identity; and AgaSK, a bifunctional enzyme with an N-terminal α-galactosidase domain and a C-terminal kinase domain (Bruel et al. 2011). According to the CAZy database, α-galactosidases from microbial organisms are grouped into GH4, GH27, GH36, GH57, GH97, and GH110 families (cazy.org.com). The three enzymes from E1 belong to the GH36 family. The *aga1, aga2*, and *agaSK* genes are expressed *in vivo* in the digestive tract of E1 mono-associated mice, suggesting a role for these enzymes in the gut (Bruel et al. 2011, Aguilera et al. 2012, Cervera-Tison et al. 2012). Activity assays confirmed that the corresponding enzymes Aga1, Aga2, and AgaSK were active on natural substrates (melibiose, raffinose, and stachyose) (Bruel et al. 2011, Cervera-Tison et al. 2012, Lafond et al. 2020). Aga1 and Aga 2 showed a preference for melibiose (Cervera-Tison et al. 2012) while AgaSK had a preference for short chain oligosaccharides (Lafond et al. 2020). AgaSK could also bind to ATP and phosphorylate sucrose provided either by raffinose hydrolysis or by the environmental medium (in the presence of ATP). AgaSK kinase activity was largely independent of the GH36 domain (Lafond et al. 2020). *Aga2* and *agaSK* (but not *aga*1) are part of gene clusters. *Aga2* is part of an unusual 6-gene operon, containing other GH genes, probably involved in extracellular and intracellular sucrose assimilation (Cervera-Tison et al. 2012). The *agaSK* gene cluster comprises three genes coding for: an ABC transporter; a raffinose-specific solute-binding protein; and a putative sucrose phosphorylase SucP of the subfamily 18 in the GH13 family (GH13_18).

GH13_18 members reversibly catalyze the reaction between sucrose and inorganic phosphate to synthesize α-D-glucose-1-phosphate (G1P) and D-fructose, which then enter microbial glycolytic pathways. In the presence of inorganic phosphate, *R. gnavus* E1 SucP showed selective phosphorylase activity on sucrose 6F-phosphate (S6FP) compared with sucrose, and was renamed *R. gnavus* sucrose 6F-phosphate phosphorylase (*Rg*SPP) (Tauzin et al. 2019). *Rg*SPP acts both in phosphorolysis of S6FP releasing α-D-glucose-1-phosphate (G1P) and α-D-fructose-6-phosphate (F6P), and in reverse phosphorolysis from G1P and F6P to S6FP. Such SPP activity had not been observed in gut bacteria before (Tauzin et al. 2019). Analysis of *Rg*SPP homologous genes in metagenomic data from various cohorts showed that the prevalence and abundance of *Rg*SPP homologous genes in the gut microbiome correlated with the geographical origin of the individuals (most likely due to diet) rather than their health status. These *in silico* findings were confirmed experimentally; mice mono-colonized with *R. gnavus* E1 showed a 5-fold increase in *Rgspp* gene expression when fed a high-fat diet (HFD) containing five times less sucrose compared with a standard diet, suggesting a close relationship between lipid and sucrose metabolism (Tauzin et al. 2019). Finally, phylogeny and synteny studies suggested that the *agaSK* gene cluster has been spread through horizontal transfer but only to a few species in the Firmicutes (Lafond et al. 2020).

Most *R. gnavus* strains have between 7 and 10 GH13 encoding genes in their genomes (see Fig. 2). GH13 is the most represented family in the gut microbiome and enzymes in this family are involved in the breakdown of starch (El Kaoutari et al. 2013). However, despite harbouring eight GH13 encoding genes, *R. gnavus* ATCC 29149 was not able to use potato soluble starch, nor maize resistant starch as sole carbon sources (Crost et al. 2018). However, this strain could use the starch degradation products released by *Ruminococcus bromii*, a specialist starch degrader, namely, maltotetraose, maltotriose, maltose, and glucose (Crost et al. 2018). Interestingly, GH13 was one of the family of enzymes upregulated when *R. gnavus* ATCC 29149 was grown on mucin as a sole carbon source (Crost et al. 2016). Together these data suggest that GH13 enzymes fulfil a distinct and yet to be defined role in *R. gnavus*.

Some *R. gnavus* strains have β-glucuronidase (GUS) activity when grown in rich media (Beaud et al. 2006). GUS belongs to GH families 1, 2, 3, 30, 79, 154, and 169 (www.cazy.org). *R. gnavus* E1 encodes one GH2 β-glucuronidase (*Rg*GUS), which has been functionally characterized (see Table 1). The *Rg*GUS encoding gene is part of an operon comprising at least four genes including a sugar transporter in the phosphotransferase system and a putative β-glucosidase. Compared with members of its closest GUS structural group, the crystal structure of *Rg*GUS has unique active site features on the L1 GUS loop (Biernat et al. 2019). *Rg*GUS exhibited catalytic efficiencies 10–100-fold lower on p-nitrophenyl-β-D-glucuronide (pNPG) than those of the L1 GUS enzymes previously characterized, and was not inhibited by two selective microbial GUS inhibitors, Inhibitor 1 and UNC10201652, which are potent against the L1 *E. coli* GUS. *Rg*GUS was also one of the slowest L1 GUS enzymes to hydrolyze the nonsteroidal anti-inflammatory drug metabolite diclofenac glucuronide. A recent study showed that L1-GUS sequences were present in 59/60 metagenomes of healthy subjects analysed; L1-GUS-encoding bacteria represented on average 2.1% of total bacteria but with very high interindividual variability (0%–24.3%) (Candeliere et al. 2022). In the colon, GUS could be involved in release of aglycone residues from plant derived glyconjugates such as lignans or flavonoids, or convert the conjugated xenobiotic and endogenous substances into unconjugated ones, thereby compromising the detoxification process (Beaud et al. 2005). However, the relevance of *R. gnavus* β-glucuronidase activity in the lower gut remains to be demonstrated.

#### Other mechanisms for *R. gnavus* interactions with mucins

The *R. gnavus* E1 strain, which lacks the ability to forage on mucin glycans, has developed alternative colonization strategies targeting mucin. *R. gnavus* E1 produces an adhesin called RadA (Maresca et al. 2021), which is part of a family of microbial surface components recognizing adhesive matrix molecules (MSCRAMMs) that are cell-wall anchored by a sortase in Gram-positive bacteria. MSCRAMMs are defined by the presence of two adjacent IgG-like folded subdomains and adhere to various substrates including extracellular matrix components such as collagen and fibrinogen. RadA preferentially binds to human immunoglobulins (IgA and IgG) and also Gal and GalNAc residues in mucin glycan chains. Moreover, the *radA* gene is transcribed in the gut of monoxenic animals, but not when E1 is grown in rich medium, suggesting a role of this adhesin in gut colonization (Maresca et al. 2021). In addition, *R. gnavus* E1 induces transcription of several mucin and GT genes in the colon of E1 mono-colonized mice compared with germ-free mice (Graziani et al. 2016). Similar results were obtained *in vitro* when mucin-secreting HT29-MTX cells were treated with the spent medium from E1 culture; this effect is likely to be mediated by a small peptide-like substance which remains to be characterized (Graziani et al. 2016).

*R. gnavus* ATCC 29149 and 35913 have been studied for their ability to forage on mucin glycans through their unique sialic metabolism pathway (see above and Crost et al. 2016). In addition, the carbohydrate binding module belonging to family 40, CBM40, which is part of *R. gnavus* ATCC 29149 GH33 intramolecular *trans*-sialidase, has been shown to bind, as an individual component and in a sialic-acid dependent manner, to mucus from mouse intestinal tissue, human cell lines and to purified mucins, providing evidence that CBM40 is a novel mucus adhesin (Owen et al. 2017). Using a microaerobic vertical diffusion chamber (VDC) with a coculture of T84 intestinal epithelial cells and mucus producing LS174T cells, *R. gnavus* ATCC 35913 conferred protection against enteropathogenic *E. coli* (EPEC) infection, by reducing EPEC growth and adhesion. This phenotype was not observed when the VDC contained T84 cells only or no host cells, suggesting that *R. gnavus* ATCC 35913 may be competing with EPEC for nutrient sources derived from mucus or for mucin attachment sites (McGrath et al. 2022).

## Association between *R. gnavus* and diseases

**Figure 3.**
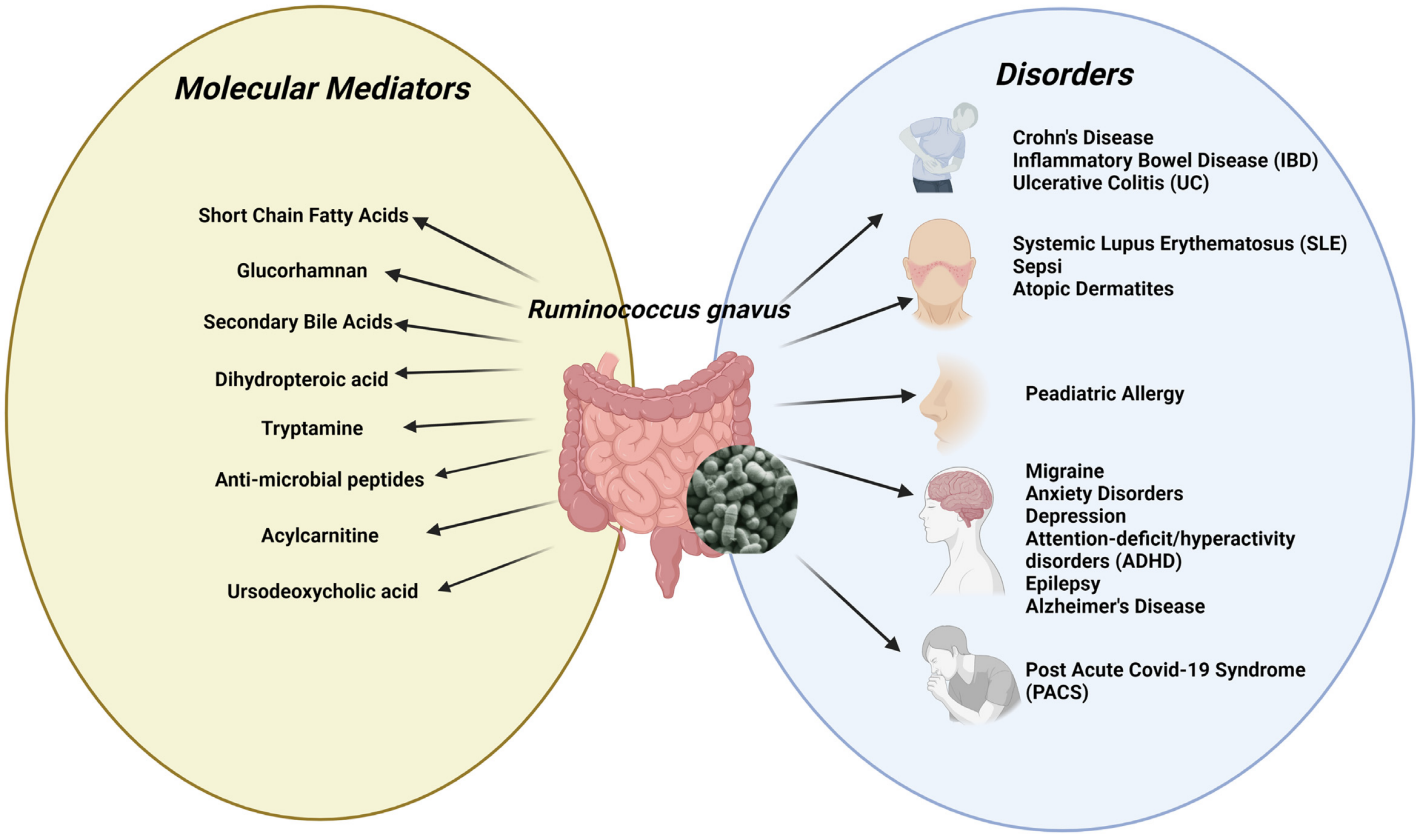
*R. gnavus* association in diseases and potential molecular mediators.

Although *R. gnavus* is part of the healthy human gut microbiota, it is disproportionately represented in gut- and non-gut-related diseases, as reviewed below (Table 2; Fig. 3). However, most correlations to date rely on analysis of faecal microbiota by 16S rRNA amplicon sequencing which provides relative abundance of bacteria at different levels (phylum, class, order, family, and genus), but does not allow resolution at the species or strain level. For example, although *Ruminococcus albus* and *R. gnavus* are genetically distinct, v3–v4 amplicon data cannot detect *R. albus* species or distinguish between the two species since most of the differences lay outside the v3–v4 hypervariable region (Zhang et al. 2021). In addition, sequencing results are highly dependent on sample preparation, storage, and bioinformatics pipelines. DNA extraction is a critical step for complete microbial cell lysis and accurate representation of the gut microbiome. These considerations are particularly relevant in Gram-positive organisms in order to identify differences at the species levels; *R. gnavus*, e.g. requires extensive bead-beating (Zhang et al. 2021). Another limitation is that, with the exception of inflammatory bowel diseases (IBD) and colorectal cancer (CRC), most metagenomics analyses are done using faecal samples, and it is therefore, not possible to relate the findings to ecological/nutrient niche-specific *R. gnavus* strains in the gut.

**Table 2.** Association of *R. gnavus* to diseases.

Disease	*R. gnavus*	Study cohort	Sampling	Reference
Crohn’s disease	Increased (*P* < .05)	Patients *n* = 40 twin pair	Stool samples, 16S sequencing	Willing et al. (2010)
	Increased (*P* = .1 × 10^−7^)	Patients *n* = 68 Healthy *n* = 55	Stool sample, DGGE	Joossens et al. (2011)
	Increased (*P* = .043)	Patients *n* = 25 Healthy *n* = 25	Stool and saliva samples, Shotgun metagenomics	Hu et al. (2021)
	Increased (FDR < 0.05)	Patients *n* = 39 Healthy *n* = 14	Stool samples, 16S sequencing	Feng et al. (2022)
	Increased	Patients *n* = 181	Human biopsies, 16S sequencing	Buisson et al. (2022)
Crohn’s disease Ulcerative colitis	Increased (*P* = 0.04)	Patients CD *n* = 26 Patients UC *n* = 20 Healthy *n* = 20	Human biopsies, 16S sequencing	Png et al. (2010)
	Increased (*P* < .05)	Patients CD *n* = 26 Patients UC *n* = 43 Healthy *n* = 14	Colonic mucosa biopsies 16S sequencing	Nishino et al. (2018)
	Increased (*P* < .05)	Patients *n* = 65 UC/CD and healthy	Stool samples, Shotgun metagenomics	Franzosa et al. (2019)
	Increased (*P* < .05)	Patients CD *n* = 39 Patients UC *n* = 70 Healthy *n* = 100	Stool samples, 16S sequencing	Shin et al. (2022)
	Increased (*P* < .001)	Patients *n* = 21	Stool sample, DGGE	Machiels et al. (2017)
Inflammatory bowel disease	Increased (*P* < .05)	Patients *n* = 56 Healthy *n* = 24	Stool samples, 16S sequencing	Sokol et al. (2018)
	Increased (*P* = .02)	Patients CD *n* = 80 Patients UC *n* = 27 Patients IBD *n* = 50 Healthy *n* = 75	Stool samples, 16S sequencing	Olbjørn et al. (2019)
	Increased (*P* < .01)	Patients P. *n* = 69 Patients UC *n* = 76 Patients CD *n* = 88 Healthy *n* = 56	Stool samples, Shotgun metagenomics	Dubinsky et al. (2021)
	Increased (*P* < .05)	Patients CD *n* = 10 Patients UC *n* = 13 Patients IBS *n* = 26 Healthy *n* = 12	Colonic mucosa biopsies and stool samples, 16S sequencing	Cipcic Paljetak et al. (2022)
Irritable bowel syndrome	Increased (*P* < .05)	Patients *n* = 80 Healthy *n* = 65	Stool samples, Shotgun metagenomics	Jeffery et al. (2020)
	Increased (*P* = 2.83 × 10^−3^)	Patients *n* = 264 Healthy *n* = 66	Stool samples, Shotgun metagenomics	Han et al. (2022a)
	Increased (*P* < .001)	Patients *n* = 87 Healthy *n* = 91	Stool samples, Shotgun metagenomics	Zhai et al. (2023)
Colorectal cancer	Increased (*P* < .001)	Patients *n* = 14 + 15	Colonic mucosa, 16S sequencing	Hong et al. (2019)
	Increased (*P* < .00065)	Patients *n* = 17 Healthy *n* = 5	Stool samples, 16S sequencing	Huang et al. (2022)
Obesity	Negative correlation (*P* < .05)	Subject *n* = 236 CHILDREN	Stool samples, 16S sequencing	Wei et al. (2021)
	Increased (*P* < .05)	Subject *n* = 84	Stool samples, 16S sequencing	Jie et al. (2021)
	Increased (*P* < .05)	Subject *n* = 41	Stool samples, Shotgun metagenomics	Yan et al. (2021)
	Increased (*P* < .0001)	Subject *n* = 5215	Stool samples, qPCR	Grahnemo et al. (2022)
Coronary artery disease	Increased (*P* = .016)	Patients *n* = 53 Subject *n* = 236 CHILDREN	Stool samples, 16S sequencing	Toya et al. (2020)
Coronary heart disease	Decreased (*P* = .043)	Patients CHC *n* = 24 Patients NAFLD *n* = 24 Healthy *n* = 24	Stool samples, 16S sequencing	Zhang et al. (2019)
Alcohol liver damage	Positive correlation (*P* = .05)	Subject *n* = 531	Stool samples, Shotgun metagenomics	Jiao et al. (2022)
Type 2 diabetes	Increased (*P* < .05)	Patients *n* = 134 Healthy *n* = 134	Stool samples, 16S sequencing	Allin et al. (2018)
	Increased (*P* < .05)	Patients *n* = 5 Healthy *n* = 5	Stool samples, 16S sequencing	Kulkarni et al. (2021)
	Positive correlation (*P* = .04)	Subject *n* = 5572	Stool samples, Shotgun metagenomics	Ruuskanen et al. (2022)
	Increased in insulin-resistant (FDR < 0.05)	Subject *n* = 258	Stool samples, 16S sequencing	Xu et al. (2023)
Diabetes	Decreased (*P* < .05)	Patients *n* = 26 Healthy *n* = 10	Stool samples, Shotgun metagenomics	Zhang et al. (2022)
Gestational diabetes mellitus	Positive correlation (*P* = .0093)	Patients *n* = 52	Stool samples, 16S sequencing	Li et al. (2021)
Covid-19 postacute syndrome	Increased (*P* < .05)	Patients *n* = 106 Healthy *n* = 68	Stool samples, 16S sequencing	Liu et al. (2022)
Covid-19	Increased (*P* < .05)	Patients *n* = 100 Healthy *n* = 78	Stool samples, Shotgun metagenomics	Yeoh et al. (2021)
General anxiety disorder	Increased (*P* < .05)	Patients *n* = 40 Healthy *n* = 36	Stool samples, 16S sequencing	Jiang et al. (2018)
Depression	Increased (72% depressed subjects)	71 subjects, *n* = 34 probiotic, *n* = 32 placebo	Stool samples, 16S sequencing	Chahwan et al. (2019)
Epilepsy	Increased and positively correlated with epilepsy (*r* = 0.541, *P* < .01)	Patients *n* = 41 Healthy *n* = 30	Stool samples, 16S sequencing	Dong et al. (2022)
Attention-deficit/hyperactivity disorder (ADHD)	Reduced (*P* < .05)	Patients *n* = 17 Healthy *n* = 17	Stool samples, Shotgun metagenomics	Wan et al. (2020)
Parkinson’s disease	Increased (*P* < .05)	Patients *n* = 165	Stool samples, 16S sequencing	Nishiwaki et al. (2022)
	Increased (*P* < .05)	Patients *n* = 103 Healthy *n* = 81	Stool samples, 16S sequencing	Lubomski et al. (2022)
Neurocognitive disorders	Increased (*P* < .05)	Patients *n* = 13 Healthy *n* = 8	Stool samples, Shotgun metagenomics	Han et al. (2022b)
Cognitive impairment	Increased (*P* < .05)	Patients *n* = 24 Healthy *n* = 23	Stool samples, 16S sequencing	Feng et al. (2021)
Migraine	Increased (*P* < .05 false discovery rate (FDR) = 0.14)	Patients *n* = 54 Healthy *n* = 54	Stool samples, Shotgun metagenomics	Chen et al. (2019)

### IBD

Crohn’s disease (CD), ulcerative colitis (UC), and pouchitis are multifactorial and chronic IBD that have consistently been associated with gut microbial dysbiosis. An increasing number of studies show a positive association between *R. gnavus* and IBD (Liu et al. 2021), although a causal relationship remains to be demonstrated. Early studies focusing on analysis of mucosal tissue from IBD patients showed an increase in abundance of mucosa-associated bacteria (Schultsz et al. 1999). For example, compared with healthy mucosa, *R. gnavus* and *R. torques* were more abundant in CD and UC mucosa while the main mucolytic bacterium *A. muciniphila* was significantly less abundant in both CD and UC mucosa (Png et al. 2010). In a follow up study, faecal samples of 68 patients with CD, 84 of their unaffected relatives and 55 matched controls were analysed by DGGE and quantified using real-time PCR. Results showed that *R. gnavus* was the only species amongst the five bacterial species underpinning dysbiosis that increased in abundance in CD (Joossens et al. 2011).

More recently, the mucosa-associated microbial community was determined by 16S rRNA gene sequencing from samples obtained from patients with IBD by gentle brushing of mucosal surfaces using endoscopic cytology brushes (43 and 26 patients with UC and CD, respectively, and 14 non-IBD controls). Abundance of the phylum Proteobacteria significantly increased while the phyla Firmicutes and Bacteroidetes significantly decreased in CD patients compared with non-IBD controls. These included significant increases in the genera *Escherichia, Ruminococcus* (*R. gnavus*), *Cetobacterium, Actinobacillus*, and *Enterococcus*, and significant decreases in the genera *Faecalibacterium, Coprococcus, Prevotella*, and *Roseburia*. Comparisons between CD and UC patients revealed a greater abundance of the genera *Escherichia, Ruminococcus* (*R. gnavus*), *Clostridium, Cetobacterium*, and *Peptostreptococcus* in CD patients, and in the genera *Faecalibacterium, Blautia, Bifidobacterium, Roseburia*, and *Citrobacter* in UC patients (Nishino et al. 2018). In another study investigating mucosa-associated dysbiosis in IBD patients, CD was characterized by colonization with adherent and invasive *E. coli* (AIEC) bacteria and was associated with a specific microbiota signature that included an increase in abundance of *R. gnavus* (Buisson et al. 2022). A study based on gut microbiota analysis of a cohort of 40 twin pairs who were concordant or discordant for CD or UC showed increased abundance of Enterobacteriaceae and *R. gnavus*, and disappearance of core bacteria such as *Faecalibacterium* and *Roseburia*, in patients with ileal CD (Willing et al. 2010). *Clostridium difficile* infection (CDI) is a common complication in IBD and has been associated with poor IBD outcomes. IBD patients with CDI had higher levels of *R. gnavus* and *Enterococcus* and lower levels of *Blautia* and *Dorea* than IBD patients without CDI (Sokol et al. 2018). The use of faecal microbiota transplantation (FMT) to treat patients with UC has generated variable outcomes. Analysis of 16S rRNA gene-based phylogenetic microarrays of faecal and mucosal samples from UC patients receiving FMT showed that a donor microbiota rich in specific members of *Clostridium* clusters IV and XIVa was associated with a sustained positive response. However, *R. gnavus* was found at high levels in donors of patients with failed FMT (Fuentes et al. 2017). *R. gnavus* was also implicated in paediatric IBD; children with ileocolitis or total colitis had more *R. gnavus* than those with colonic CD or left-sided UC in a study using 16S rRNA DNA-based analysis of bacterial abundance in faecal microbiota from 235 children younger than 18 years of age (80 with CD, 27 with UC, three IBD unclassified, 50 non-IBD symptomatic patients, and 75 healthy) (Olbjorn et al. 2019). A total of 12 species were uniquely differentially abundant and enriched in CD, including *R. gnavus, E. coli*, and *Clostridium clostridioforme* (Franzosa et al. 2019).

Recently, the gut microbiomes of Asian subjects with CD were characterized using whole genome shotgun sequencing, which enabled strain-level and metabolic pathway analyses. Gut species found to be significantly depleted in CD compared with healthy controls included *Faecalibacterium prausnitzii, Roseburia inulinivorans*, and *Alistipes senegalensis* whereas *Clostridium nexile* and *R. gnavus* were enriched. Microbial arginine and isoprene pathways were at higher relative abundance in the gut microbiome of CD patients (Hu et al. 2021). Recent analysis of the faecal microbiota of Korean patients with IBD (70 with UC, 39 with CD) and 100 healthy control individuals using Illumina MiSeq revealed that *R. gnavus* was a biomarker for prognosis in CD (Shin et al. 2022). Whole genome sequencing of *R. gnavus* isolates coupled with metagenomic analyses from 20 IBD patients and 12 controls revealed two phylogenetically distinct *R. gnavus* strains that appeared differentially enriched in IBD (Hall et al. 2017), highlighting the importance of analysing the effect of *R. gnavus* on IBD at the strain level. This study also revealed IBD-specific, strain-specific genes involved in oxidative stress responses, adhesion, iron-acquisition, and mucin utilization, potentially conferring *R. gnavus* with an adaptive advantage in the gut of patients with IBD (Hall et al. 2017). Several mechanisms underpinning the association between *R. gnavus* and IBD have been proposed following investigations in mouse models (see section on the 'Molecular mediators underpinning the effect of *R. gnavus* on health and disease'). In addition, daily changes in circulating neutrophil, lymphocyte, and monocyte counts have been analysed alongside >10 000 longitudinal microbiota samples from cancer patients receiving haematopoietic cell transplantation and stem cell engraftment following chemotherapy; this revealed a negative association between absolute *R. gnavus* group abundance and lymphocytes rates, and a positive association between lymphocyte counts and *R. gnavus* group growth rates suggesting that *R. gnavus* may drive high neutrophil-to-lymphocyte ratios that are broadly characteristic of poor disease outcomes in IBD (Schluter et al. 2020). Enrichment of *R. gnavus* was positively correlated with psychological score in CD patients and negatively correlated with two secondary bile acids, taurodeoxycholic acid (TDCA) and taurolithochoic acid (TLCA), directly related to psychological scores in these patients (Feng et al. 2022).

Pouchitis is a common postoperative complication of UC that develops after proctocolectomy and ileal–pouch–anal anastomosis (IPAA) and is characterized by inflammation of the previously normal small intestine comprising the pouch. The faecal microbiota of 85 participants (37 UC, 15 healthy UC pouches, 15 pouchitis, and 18 healthy participants) was analysed by pyrosequencing of 16S ribosomal DNA. The results showed that aggravation of UC was characterized by a gradual decrease in diversity and abundance of butyrate-producing bacteria and *Bacteroides*, and an increase in *Escherichia–Shigella*; also *R. gnavus* was observed in pouchitis and related to multiple infection pathways (Gao et al. 2022). In another study, 208 faecal metagenomes from 69 patients with a pouch (normal pouch and pouchitis) were compared with publicly available metagenomes of patients with CD (*n* = 88), UC (*n* = 76), and healthy controls (*n* = 56). Patients with pouchitis presented with the largest number of alterations in species, metabolic pathways, and enzymes, which was correlated with intestinal inflammation. *R. gnavus* strains were highly enriched in pouchitis. The representation of butyrate and secondary bile acid biosynthesis pathways was decreased in IBD phenotypes, and particularly in pouchitis. Pathways such as amino acid biosynthesis and degradation of aromatic compounds and sugars, encoded by members of the Enterobacteriaceae, were enriched in pouch and CD patients but not in UC patients (Dubinsky et al. 2021). The presence of *R. gnavus, B. vulgatus*, and *C. perfringens* and absence of *Blautia* and *Roseburia* in faecal samples of patients (*n* = 21) with UC undergoing IPAA but before colectomy surgery, was associated with a higher risk of pouchitis after surgery; this suggests that the risk of pouchitis can be predicted based on the faecal microbial composition before surgery (Machiels et al. 2017). Taken together, studies point consistently towards a role for *R. gnavus* as a biomarker of IBD, but causality remains to be demonstrated.

### Irritable bowel syndrome

Irritable bowel syndrome (IBS) is a chronic and heterogeneous disorder that affects around 6% of the population. The pathophysiology of IBS is multifactorial and thought to include an altered gut microbiota. Bacterial sequencing analysis of stool samples and colon biopsies from IBS patients and healthy individuals as well as samples from a mouse model of IBD with 2,4,6 trinitrobenzene sulfonic acid (TNBS) induced colitis, showed that the severity of symptoms and the gravity of inflammation are most likely to be linked to an alteration in mucus-associated bacteria such as *R. gnavus* (Kozik et al. 2019). Diagnosis and determination of subtypes is based on symptoms of IBS. Shotgun and 16S rRNA sequencing of faeces from 80 patients with IBS (Rome IV criteria; 16–70 years old) and 65 matched individuals without IBS (control individuals) showed that *R. gnavus* and other species in the Lachnospiraceae, were significantly more abundant in IBS based on pairwise comparisons (Jeffery et al. 2020). *R. gnavus* was significantly more abundant in patients with diarrhoea-predominant IBS (IBS-D). This was associated with peripheral 5-hydroxytryptamine (5-HT) and severe symptoms, as also shown in *R. gnavus* mono-colonized mice (Zhai et al. 2023) (see section on the 'Molecular mediators underpinning the effect of *R. gnavus* on health and disease'). By integrating multiple data layers, purine metabolism was identified as a novel host–microbial metabolic pathway in IBS (Mars et al. 2020). Metabolomic and metagenomic studies using stool and serum samples of patients with IBS showed that enrichment of *R. gnavus* was strongly associated with low levels of dihydropteroic acid, an important intermediate in folic acid synthesis, contributing to metabolic dysregulation in IBS pathology (Han et al. 2022a). The role of *R. gnavus* (amongst other species) as a potential biomarker for discriminating disease phenotypes was further confirmed in a 16S rRNA gene sequencing-based comparison of gut faecal and mucosa microbiota in newly diagnosed, treatment-naïve adults with IBD or IBS (13 UC, 10 CD, and 26 IBS) and in a healthy group (Cipcic Paljetak et al. 2022).

### CRC

Several metagenomics studies have linked CRC with a significant alteration in composition of the intestinal mucosa-associated microbiota compared with control subjects. Aberrant crypt foci (ACF) are the earliest morphologically identifiable lesions in the colon of patients with CRC. Profiling colon-adherent bacteria from individuals undergoing a screening or surveillance colonoscopy showed significantly greater heterogeneity in the ACF bacterial microbiome profiles compared with normal mucosa; mutations in the KRAS oncogene were positively correlated with *R. gnavus*, indicating a potential relationship between specific colon-associated bacterial species and somatically acquired CRC-related mutations. These findings suggest that perturbations in the normal adherent mucosal microbiota may constitute a risk factor for early neoplasia, demonstrating the potential impact of mucosal dysbiosis on the tissue microenvironment and behaviour of ACF that may facilitate their progression towards more advanced forms of neoplasia (Hong et al. 2019). In a recent study, 16S amplicon sequencing of the gut microbiota from CRC patients before and after treatment showed that the *R. gnavus* group was implicated in the adipocytokine signalling pathway and peroxisome pathway (Huang et al. 2022).

### Metabolic diseases

*R. gnavus* is reproducibly associated with several features of metabolic syndrome in humans, including an increase in % body fat, as supported by prior studies reviewed below and a seminal publication based on analysis of a large discovery cohort (*n* = 2875) and two replication cohorts (*n* = 999 and *n* = 1341) of participants aged 20–94 years from Norway (Grahnemo et al. 2022). *R. gnavus* was present in 33.7% of participants in the combined cohort, and its presence was robustly associated with several features of metabolic syndrome including an increase in fat mass, waist circumference, serum triglycerides, C-reactive protein (CRP), HbA1c, and a decrease in HDL after adjustment for multiple confounders. These associations were similar in both men and women and in participants younger and older than the median age of 59.6 years when evaluated in stratified analyses (Grahnemo et al. 2022).

#### Obesity

Abdominal obesity significantly increases the risk of metabolic syndrome and cardiovascular disease. In the cross-sectional Multiethnic Cohort Adiposity Phenotype Study (812 men, 843 women, 60–77 years), analyses of the faecal microbiota of participants by 16S rRNA gene sequencing implicated *R. gnavus* in associations with energy-adjusted dietary inflammatory index (E-DII) and adiposity phenotypes (Lozano et al. 2022). In a study of 41 people with normal body mass index (BMI), *R. gnavus* was one of the 16 gut microbial species that had a strong correlation with visceral fat and were positively correlated with metabolic indicators (Yan et al. 2021). This is consistent with earlier studies showing that: *R. gnavus* (MGS0160) was significantly enriched in obese individuals and decreased in abundance during weight loss (Jie et al. 2021); and that there were correlations between *R. gnavus* and high BMI (Verdam et al. 2013). It is also of note that according to a recent study investigating whether preoperative gut microbiota composition could predict responses to bariatric surgery, *R. gnavus* was found to be part of the non-responder group according to the percentage of excess weight loss 3 months after surgery. Before surgery, the non-responder group showed an enrichment in pathways involved in the biosynthesis components of LPS O-antigens (Gutierrez-Repiso et al. 2022). Analysis of the abundance of 50 prevalent gut microbes in stool samples of participants (aged 20–94) of the cross-sectional, population-based Norwegian Trøndelag Health (HUNT) study using species-level qPCR confirmed direct associations between *R. gnavus* and an increase in fat mass, waist circumference, serum triglycerides, and CRP (Grahnemo et al. 2022). This study also showed an additive effect of incorporating information on the presence or absence of *R. gnavus* in the prediction of metabolic traits based on low or high BMI genetic risk score. However, a causal relationship between presence of *R. gnavus*, fat mass, and BMI remains unknown. It has been suggested that some of these effects involve changes in intestinal mucus function and permeability. However, although animal studies showed that *R. gnavus* was enriched in obese rats (Petriz et al. 2014) and in mice fed a HFD (Wu et al. 2021c), there was no evidence that *R. gnavus* had a role in mucus penetrability in ob/ob mice (Schroeder et al. 2020). Furthermore, colonic permeability was not associated with plasma CRP or faecal calprotectin levels in obese individuals with higher levels of *R. gnavus* (Verdam et al. 2013). Recent animal studies suggest that *R. gnavus* influences host metabolites (see also section on the 'Molecular mediators underpinning the effect of *R. gnavus*on health and disease'). In HFD mice, *R. gnavus* affected signalling pathway and downstream lipid metabolism in the liver, specifically decreased FGF21 expression, and increased hepatic triglyceride levels, serum levels of total cholesterol and LDL, all contributing to metabolic disorder (Wu et al. 2021c). In gnotobiotic mice, addition of *R. gnavus* ameliorated growth and metabolic abnormalities in animals receiving FMT from undernourished infants aged 6–18 months (Blanton et al. 2016); again, the presence of *R. gnavus* affected metabolic phenotypes in the liver, indicating a role for *R. gnavus* in lipid metabolism in the gut–liver axis. Notably, the associated decrease in acylcarnitines (C5–C16) in the liver suggests an impact of *R. gnavus* on host metabolic machinery that drives amino acids away from oxidation in favour of protein synthesis and lean mass formation (Blanton et al. 2016). Recent evidence suggests that gut microbiota and cardio-metabolic status are associated. An intervention study of adults with prediabetes and IR demonstrated that *R. gnavus* was positively correlated with hepatic IR (Zhang et al. 2022). Supplementation with (poly)phenol-dense red raspberries decreased *R. gnavus* and reduced hepatic insulin-resistance as well as total and LDL cholesterol in the plasma, suggesting that dietary interventions targeting *R. gnavus* may be used to promote metabolic health in adults with prediabetes-IR (Zhang et al. 2022). These studies showed consistent positive associations between *R. gnavus* and obesity-related parameters. However, a cross-sectional study involving 236 children aged 6–9 years showed significant negative correlations between *R. gnavus* and total and regional body fat while OTUs belonging to the genera *Blautia* and *Romboutsia* exhibited positive correlations with body fat measures and obesity-related parameters (Wei et al. 2021). These discrepancies may be due to the different age range and diet of participants but variations due to different microbiome profiling approaches cannot be excluded.

#### Coronary artery and liver diseases

Alterations in gut microbiome composition has been linked to cardiovascular diseases. To identify specific bacterial communities associated with coronary artery diseases (CAD), a case-control study analysed the faecal microbiota of 53 advanced CAD patients and 53 age-, sex-, race-, and BMI-matched controls using 16S rRNA sequencing. CAD patients exhibited a decreased richness and evenness in their gut microbiome. *R. gnavus* was amongst five taxa that showed more than a two log-fold increase in relative abundance and, after adjustment for coronary risk factors (diabetes mellitus and dyslipidaemia), this was associated with the presence of advanced CAD (Toya et al. 2020).

Patients suffering from coronary heart disease (CHD) complicated with nonalcoholic fatty liver disease (NAFLD) have worse cardiac function and clinical outcomes than patients with CHD only. In a study investigating the contribution of the gut microbiota in this disease, CHD–NAFLD patients exhibited an increase in BMI, uric acid and triglyceride, which was paralleled with a significant reduction in the abundance of *R. gnavus* and *Bacteroides dorei* compared with CHD patients. It has been proposed that alterations in *R. gnavus* levels may affect the degree of metabolic disorder contributing to worse clinical outcomes and disease progression than in CHD patients (Zhang et al. 2019). Alcoholic liver damage is usually identified by elevated liver transaminase. A recent study investigating the role of the gut microbiome in individual susceptibility to alcoholic liver injury showed that *R. gnavus* exhibited the strongest positive association with alanine aminotransferase (ALT) and aspartate aminotransferase (AST), and contributed to liver inflammation (Jiao et al. 2022).

#### Diabetes

Differential alterations in gut microbiota and chronic low-grade inflammation play a critical role in the development of type 2 diabetes (T2D). A small sized pilot study using Illumina sequencing (MiSeq platform) of the V3 region of the 16S rRNA gene of faecal samples from five patients with T2D showed a higher abundance of *R. gnavus* as well as *Lactobacillus ruminis, Bacteroides caccae, Butyricimonas*, and *Collinsella aerofaciens*; and a lower abundance of anti-inflammatory bacteria such as *F. prausnitzii* and *Butyrivibrio* (Kulkarni et al. 2021). However, recent studies suggest that the T2D medication metformin alters the composition and functional potential of gut microbiota, thereby interfering with the diabetes-related microbial signatures. Long-term associations between gut microbiome composition and incident T2D were studied in a representative population cohort (432 cases of incident diabetes occurred over the median follow-up of 15.8 years) and identified *R. gnavus* as one of the four species consistently associated with incident diabetes (Ruuskanen et al. 2022). Similarly, analysis of the gut microbiota of 134 Danish adults with prediabetes showed that the two OTUs that differed the most were a member of the order Clostridiales and *A. muciniphila*, which both displayed lower abundance amongst individuals with prediabetes while *R. gnavus* abundance increased (Allin et al. 2018). A recent study investigating microbiome features based on the unsupervised stratification of 258 patients with T2D showed that *R. gnavus* was enriched in a cluster characterized by a moderate level of blood glucose, serious insulin resistance, and high levels of cholesterol and triglyceride (Xu et al. 2023). Amelioration of T2D after bariatric surgery also depended on changes in the gut microbiota. In T2D rats, modified jejunoileal bypass increased abundance of *R. gnavus* and *E. coli* as well as levels of serum glycine, histidine, and glutamine; and decreased abundance of *Prevotella copri* and levels of serum branched chain amino acids, which are significantly related to improvement in islet β-cell function (Tan et al. 2021). These results point towards a role for the microbial amino acid metabolism pathway in T2D as also observed in animal models of malnutrition (as described above). Correlation analysis between gestational diabetes mellitus (GDM) and gut microbial composition in pregnant women at different stages of pregnancy (28–36 weeks and 36–41 weeks) showed that *R. gnavus* was positively correlated with fasting blood glucose during late pregnancy in patients with GDM (Li et al. 2021). A study on diabetic peripheral neuropathy (DPN) showed that glycine ursodeoxycholic acid and tauroursodeoxycholic acid were positively correlated with the *R. gnavus* group and *Phascolarctobacterium* richness (Wang et al. 2020).

### Allergy, immunity, and inflammation

Gut microbiome dysbiosis occurs in allergic children, with *R. gnavus* emerging as a main player in paediatric allergy. Several studies have shown that the gut microbiota of infants with atopic dermatitis (AD), a common inflammatory skin disease, differs from that of infants without AD. Recently, the functional profile of the gut microbiome from 40 6-month-old infants (20 control subjects and 20 patients with AD), randomly selected from a cohort of 129 infants, was analysed by whole-metagenome sequencing, revealing differences in functional genes related to immune development. Genes significantly associated with stunted immune development could be due to reduced colonization of *A. muciniphila, R. gnavus*, and Lachnospiraceae bacterium 2_1_58FAA in the AD group compared with the control group (Lee et al. 2018). This was further investigated in preclinal studies supporting the protective role of *R. gnavus* in AD mouse models (see section on the 'Molecular mediators mediating the effect of *R. gnavus* on health and disease'). The link between allergic diseases and nutrition during childhood has been highlighted in RCTs. For example, in the German Infant Nutritional Intervention RCT trial, 4-month-old breastfed infants with a family history of atopic eczema remained at decreased risk of atopy 6 years later when fed EHF compared with CMF in the first 4–6 months (von Berg et al. 2017). Since *R. gnavus* increased in EHF (above), these data are consistent with a protective effect of *R. gnavus* in AD.

In contrast, a higher abundance of *R. gnavus* (and *F. prausnitzii*) was observed in children affected by food or respiratory allergies (De Filippis et al. 2021). In a study of a prospective twin cohort, increased abundance of *R. gnavus* in the faecal microbiota was observed before the onset of allergic manifestations and was associated with respiratory allergies or respiratory allergies coexistent with atopic eczema (Chua et al. 2018). In mice, endogenous *R. gnavus* grew rapidly in abundance after sensitization and challenge with ovalbumin, and addition of *R. gnavus* ATCC 29149 led to airway hyper-responsiveness and airway inflammation (asthma), characterized by expansion of T-helper 2 cells in the colon and lung, and infiltration of colon and lung parenchyma by eosinophils and mast cells (Chua et al. 2018).

Abundance of *R. gnavus* was also significantly increased in systemic lupus erythematosus (SLE), the most common form of lupus, a chronic autoimmune disease that causes inflammation in connective tissues. Intestinal expansions of *R. gnavus* populations in SLE was directly proportional to overall disease activity and most pronounced in those with lupus nephritis. Patients with lupus nephritis had more faecal sIgA-coated *R. gnavus* bacteria than healthy controls and elevated levels of serum IgG, predominantly in response to *R. gnavus* strain-specific cell wall lipoglycan antigens (Azzouz et al. 2019). Increased levels of *R. gnavus* have also been linked to spondyloarthritis, a family of inflammatory rheumatic diseases (Breban et al. 2017) but information is lacking regarding the mechanisms underpinning these associations. There is also observational evidence for higher levels of *R. gnavus* in the gut microbiome of patients with COVID-19 (without controlling for antibiotics) (Yeoh et al. 2021) and with long-term complications of COVID-19 (Liu et al. 2022), which may be due to gut–lung translocation of bacteria and/or regulation of immunity and inflammation.

### Infection and sepsis

There have been reported cases of infections attributed to *R. gnavus* including blood malignancies and peripheral infections such as urogenital infections. For example, a pure growth of *R. gnavus* was cultured from bilateral tubo-ovarian abscesses from a young female with concurrent deep infiltrating endometriosis and evidence of pelvic inflammatory disease (Veale et al. 2021). Advances in identification methods such as matrix-assisted laser desorption ionization-time of flight mass spectrometry (MALDI-TOF MS) analysis allow direct identification from blood cultures and have resulted in more frequent identification of *R. gnavus* from clinical specimens. However, accurate identification by 16S rRNA sequencing is often required for confirmation. Cases of bloodstream infection by *R. gnavus* have been associated with diverticular disease (Hansen et al. 2013), septic arthritis (Titecat et al. 2014), gall bladder perforation (Kim et al. 2017), intestinal perforation in a patient undergoing chemotherapy for multiple myeloma and cancer of the sigmoid colon (Fontanals et al. 2018), and faecal peritonitis secondary to small-bowel herniation and perforation (Lefever et al. 2019). These data suggest that *R. gnavus* infection and potential pathogenicity is associated with damage to the GI tract. In a recent case of bloodstream infection by *R. gnavus*, the patient was treated with intravenous meropenem and ceftriaxone based on antimicrobial susceptibility tests (Fan et al. 2022).

### Neurological disorders

Emerging evidence suggests that microbial dysbiosis can contribute to the onset and progression of neurocognitive disorders such as schizophrenia, depression, bipolar disorder, anxiety, and dementia (de la Fuente-Nunez et al. 2018, Halverson and Alagiakrishnan 2020, Socala et al. 2021). It has been suggested that diet, lifestyle, genetic factors, and external stressors ‘cooperate’ to modulate gut microbiota composition and consequently the brain’s responses (Oriach et al. 2016, Novotny et al. 2019). A growing number of metagenomics studies have reported altered levels of *R. gnavus* in patients suffering neurological disorders as described below and in Table 2.

Despite the limited size of the study, a marked reduction in intestinal microbial richness and diversity was associated with increased *R. gnavus* abundance in patients suffering general anxiety disorders compared with healthy individuals (Jiang et al. 2018). An 8-week clinical trial testing the role of probiotics on treatment of the symptoms of depression showed that, although a reduction in symptoms was observed in both probiotics and placebo groups, no change in the gut microbiota was detected between the groups. However, the relative abundance of *R. gnavus* increased in patients in the severe Beck Depression Index range of depression compared with both those in the mild/moderate range and in nondepressed groups; this shows a significant and positive correlation between *R. gnavus* and the severity score on the depression anxiety stress scale (Chahwan et al. 2019). In a recent study investigating the contribution of the gut microbiota in patients suffering with brain epilepsy, a neurological disease characterized by a predisposition to seizures, *R. gnavus* was significantly positively correlated with occurrence of epilepsy compared with the control group (Dong et al. 2022).

The contribution of the gut microbiota–brain axis has also been investigated in autism spectrum disorders (ASD) and attention-deficit/hyperactivity disorders (ADHD); it has been shown that several GI disorders, like constipation and abdominal pain, negatively affect the lifestyle of subjects with these disorders (Buie et al. 2010, Ming et al. 2018). However, to date, associations between the gut microbiota and the pathophysiology of ASD and ADHD have been inconsistent across studies (Ho et al. 2020, Wang et al. 2022). In a recent study where faecal samples of ADHD children and healthy controls (matched for age) were shotgun sequenced, *R. gnavus* was significantly less abundant in the ADHD group compared with the control group (Wan et al. 2020). This alteration was associated with differences in the metabolic pathways of neurotransmitters (e.g. serotonin and dopamine), which may contribute to ADHD symptoms (see also section on the 'Molecular mediators underpinning the effect of *R. gnavus* on health and disease'). An earlier study of 23 children with ASD compared with controls showed increased abundance of *Sutterella* spp. and *R. torques* in faeces of children with ASD, while absolute and relative numbers of *R. gnavus* did not differ significantly between groups (Wang et al. 2013).

The correlation between gut microbiota and neurodegenerative diseases such as Alzheimer’s disease (AD) and Parkinson’s disease (PD) is gaining attention due to the ageing population revealing the contribution of microbial metabolites in progression of brain pathologies (De-Paula et al. 2018, Peterson 2020). In AD patients, a main feature is accumulation of amyloid β-protein (Aβ) in brain regions involved in memory (Nishiwaki et al. 2022). In a study using an AD transgenic mouse model, overexpression of Aβ protein significantly impacted the gut microbial community, characterized by decreased growth of *R. gnavus* and other species compared with the control group due to antimicrobial activity (Dos Santos Guilherme et al. 2020). In a recent study, the gut microbial population of PD patients, grouped according to disease severity, was investigated to develop microbiota-based computational models that could predict disease progression in subsequent years. The study showed that *R. gnavus* alongside *Faecalibacterium* tended to be higher in relative abundance in patients suffering of dementia with Lewy bodies (Nishiwaki et al. 2022). These results were in line with another study on the gut microbiota of PD patients versus healthy controls, where abundance of the *R. gnavus* group increased significantly compared with the control group (Lubomski et al. 2022).

Enrichment of *R. gnavus* was also observed in a cohort of elderly people vulnerable to neurocognitive disorders (Han et al. 2022b) and in CD patients suffering with psychological symptoms (Feng et al. 2022). *R. gnavus* abundance was also increased in patients with post-stroke cognitive impairment, compared with healthy control individuals; this enrichment was negatively correlated with the results of adult cognitive impairment tests (Feng et al. 2021). A shotgun metagenomics analysis of faecal samples from elderly women suffering from migraines versus healthy subjects (matched for age and BMI), identified *R. gnavus* as amongst the most significantly enriched species in the migraine group (Chen et al. 2019).

Taken together, these studies indicate an increase in *R. gnavus* in neurological disorders although a causal relationship remains to be demonstrated. There is a tendency in the literature to associate the presence of mucosa-associated or mucolytic bacteria with increased permeability of the gut barrier and/or inflammation, leading to leakage of bacterial metabolites or molecules triggering inflammation and signalling to the brain (Herath et al. 2020). However, it should be noted that there is no evidence that *R. gnavus* can physically alter the mucosal barrier. In contrast to species such as *Bacteroides thetaiotaomicron* or *A. muciniphila*, which can degrade full mucin glycan chains due to their extensive mucin-glycan degrading GH arsenal (Collado et al. 2007, Martens et al. 2008, Cirstea et al. 2020a,b, Kostopoulos et al. 2021), the ability of *R. gnavus*’ to ‘degrade’ mucin glycans is strain-specific and confined to foraging glycan epitopes away from the epithelium barrier (see section on the 'Adaptation mechanisms of *R. gnavus* to the gut') (Bell and Juge 2021).

## Molecular mediators underpinning the effect of *R. gnavus* on health and disease

### Immunity and inflammation in mouse models

Despite the positive association between *R. gnavus* and IBD (see section on the 'Association between *R. gnavus* and diseases'), preclinical studies showed that supplementation of humanized microbiota mice with *R. gnavus* ATCC 29149 led to enhanced expression of Reg3γ (Surana and Kasper 2017); this is an important antimicrobial peptide produced in gut epithelial Paneth cells that helps maintain spatial segregation between the epithelium and the microbiota and promotes gut homeostasis (Vaishnava et al. 2011). TNBS administration in mice (to chemically induce colitis) led to decreased *R. gnavus* (and *A. muciniphilia*) abundance in caecal contents, as determined by 16S rRNA gene sequencing (Alrafas et al. 2019). This was reversed following treatment with resveratrol, a natural polyphenol. FMT from resveratrol-treated mice increased resistance to TNBS-induced colitis in recipient mice by inducing Tregs while suppressing inflammatory Th1/Th17 cells, supporting a protective role for resveratrol-induced microbiota against colitis (Alrafas et al. 2019). In line with this study, orally administered *R. gnavus* ATCC 29149 decreased the severity of inflammation in mouse models of colitis (Grabinger et al. 2019).

At the molecular level, *R. gnavus* strain ATCC 29149 harbours glucorhamnan on the cell surface; this polysaccharide has demonstrated proinflammatory properties *in vitro* through induction of inflammatory cytokine secretion (TNF-α), which is also dependent on binding to TLR4 receptors, as shown using bone marrow dendritic cells (BMDCs) isolated from wild-type or TLR4 knockout mice (Henke et al. 2019). The rhamnose backbone of glucorhamnan is made up of 1,2- and 1,3-linked rhamnose units, and the sidechain has a terminal glucose linked to a 1,6-glucose. Chemical synthesis of the pentasaccharide repeating unit of the O-specific polysaccharide has been achieved (Haynie et al. 2021, Pal et al. 2021). The synthesized pentasaccharide repeat of glucorhaman was sufficient to induce an immune response and release of inflammatory cytokines such as TNF-α and IL-6 from BMDCs through TLR4 receptor recognition (Haynie et al. 2021). A follow up study showed that, in addition to glucorhamnan, some strains of *R. gnavus* including ATCC 29149, display a large capsular polysaccharide (CPS) with a molecular weight of more than 100 kDa on their cell surface. Monosaccharide analysis revealed the capsule to be composed of glucose, N-acetyl-quivosamine and GalNAc (Henke et al. 2021), although structural composition remains to be determined. It has been proposed that this polysaccharide is tolerogenic as *R. gnavus* CPS-positive strains did not induce an immune response in *vitro* or *in vivo* (Henke et al. 2021). This work identified inflammatory and anti-inflammatory factors in *R. gnavus* contributing to strain-specific immunomodulatory properties and underscore the importance of studying *R. gnavus* host immune response at the strain level.

The capacity of *R. gnavus* strains to elicit anti- or proinflammatory responses is also dependent on host physiological status, as elegantly demonstrated in a mouse study investigating the role of Paneth cell lysozyme (Lyz1) in colitis (Yu et al. 2020). Paneth cell metaplasia in the descending colon and rectum, and aberrant lysozyme production, are hallmarks of IBD pathology. Ectopic Lyz1 production in colonic epithelium was able to suppress lysozyme-sensitive bacteria such as *R. gnavus* ATCC 29149 and exacerbated colitis. Lyz1^−/−^ mice were protected from experimental colitis while antibiotic-treated Lyz1^−/−^ mice developed severe colitis. However, transfer of Lyz1*^−/−^* microbiota to lysozyme-intact hosts was not sufficient to transfer protection. *R. gnavus* colonization ameliorated colitis in antibiotic-treated Lyz1^−/−^ mice but exacerbated colitis in antibiotic-treated WT mice, suggesting that the *R. gnavus*-mediated immune response *in vivo* was dependent upon lysozyme processing as supported by *in vitro* data showing that lysozyme-processed *R. gnavus* elicited a range of inflammatory cytokines. In Lyz1^−/−^ mice, *R. gnavus* elicited a type 2 immune response causing epithelial reprograming and enhanced anticolitogenic capacity while *R. gnavus* drove proinflammatory responses in Lyz1^+/+^ mice (Yu et al. 2020).

Recent evidence has indicated that mitochondrial function may be an important factor in the pathogenesis of UC. MCJ is a mitochondrial inner membrane protein and a natural inhibitor of respiratory chain Complex I. Colitis-induced MCJ-deficient mice showed higher expression of *Myd88* and *Tlr9*proinflammatory genes, and disease severity was associated with distinct microbiota metabolism and composition (including *R. gnavus*) and elevated IgA levels (Pascual-Itoiz et al. 2020). This association may be linked to the ability of *R. gnavus* to bind directly to a variety of immunoglobin A (IgA) in a ‘superantigen’-like mode; *R. gnavus* is known to be highly IgA coated regardless of IgA target (Bunker et al. 2019).

As mentioned above (see section on the 'Association between *R. gnavus* and diseases'), disproportionately high levels of *R. gnavus* have been reported in infants with AD and respiratory allergies compared with healthy subjects. Studies in mice showed that orally administered *R. gnavus* ATCC 29149 significantly reduced AD-associated parameters (i.e. transepidermal water loss, clinical score, total serum immunoglobulin (Ig) E level, OVA-specific IgE level, and skin inflammation) in a mouse model of AD (Ahn et al. 2022). Administration of *R. gnavus* ATCC 29149 also resulted in significant downregulation of T helper 2-related cytokine mRNA and upregulation of interleukin (IL)-10 and Foxp3 in the skin. The population of CD4 + FOXP3 + T cells in mesenteric- and skin-draining lymph nodes and butyrate levels in the caecum increased in *R. gnavus*-administered AD mice. This study indicates that immune modulation by orally administered *R. gnavus* may alleviate AD symptoms through enhancement of regulatory T-cell counts and SCFA production in AD mice. In mouse models of allergy following ovalbumin sensitization/challenge, *R. gnavus* ATCC 29149 administration by oral gauge intragastric intubation, led to secretion of cytokines (interleukin [IL] 25, IL33, and thymic stromal lymphopoietin) by colon tissues, which activated type 2 innate lymphoid cells (ILCs) and dendritic cells to promote differentiation of T-helper 2 cells and production of their cytokines (IL4, IL5, and IL13). This led to infiltration of the colon and lung parenchyma by eosinophils and mast cells (Chua et al. 2018). The ability of *R. gnavus* to modulate the immune response was also demonstrated in gnotobiotic mice. Germ-free mice mono-colonized with *R. gnavus* ATCC 29149 had fewer IL22-expressing CD4 + T cells and ILCs along the Gl tract and in lymphatic organs than germ-free or specific-pathogen-free mice (Geva-Zatorsky et al. 2017). These studies clearly show that the capacity of *R. gnavus* strains to elicit anti- or proinflammatory responses is dependent on the strain and the disease model.

### SCFAs

The gut microbiota influences host physiology through production of a wide range of metabolites (Krautkramer et al. 2021). These include SCFAs and neurotransmitters, folate, indoles, secondary bile acids, and trimethylamine-N-oxide (McCarville et al. 2020, Fan and Pedersen 2021). These metabolites can act locally on the GI tract homeostasis and gut barrier or have long-term effects on the host by interacting with G-protein coupled receptors and modulating downstream pathways (Schroeder and Backhed 2016, Lavelle and Sokol 2020). They also play a role in regulating bacteria–bacteria interactions within the gut microbiota community (Cani 2018). It is suggested that the role of the gut microbiota in mood disorders occurs through regulation and release of microbe-derived neurotransmitters and metabolites, such as serotonin, GABA, and tryptophan. These metabolites can pass through the intestinal barrier and enter the circulatory system to reach the brain through the blood–brain barrier (Moos et al. 2016, Oriach et al. 2016, Leeming et al. 2019). Integration of host-, diet-, and microbial-derived signals contributes greatly to development of a metabolic profile that influences host physiology. Despite intricate cross-talk between host and microbes, new advanced technologies have identified multiple actionable microbial targets that are relevant for host health.

SCFAs such as acetate, butyrate, and propionate are metabolic products of the degradation of complex carbohydrates by gut bacteria and they represent the most studied metabolites in relation to their important role as an energy source for colonocytes and in the maintenance of gut barrier and immunity (Rooks and Garrett 2016, Gu et al. 2021, Krautkramer et al. 2021). *R. gnavus* ATCC 29149 was first characterized for its ability to produce acetate, formate, ethanol, and a small amount of lactate, but not butyrate, in peptone–yeast extract glucose broth culture (Moore et al. 1976). It was later shown that *R. gnavus* ATCC 29149 produced propanol and propionate when grown on fucosylated substrates including mucins and HMOs (Crost et al. 2013). Low concentrations of propanol were also detected at late stages of *R. gnavus* growth in glucose or in co-culture with the starch degrader *R. bromii* L2-63. Interestingly, propanediol, a precursor of propionaldehyde, is also present in these conditions. Propionaldehyde can be converted into propanol, by propanol dehydrogenase (PduQ) or into propionyl-CoA, a precursor of propionate, by CoA-dependent propionaldehyde dehydrogenase (PduP). Both pduP and pduQ genes are present in the *R. gnavus* ATCC 29149 genome, suggesting that both propanol and propionate can be produced via the propane-1, 2-diol pathway in this bacterium (Reichardt et al. 2014, Crost et al. 2018). Acetate, formate, and ethanol were produced in increasing amounts by *R. gnavus* ATCC 29149 when in monoculture with glucose or co-culture with *R. bromii* on starch, while neither butyrate or propionate were detected in the growth conditions tested (Crost et al. 2018). SCFAs have been associated with the effects of *R. gnavus* in animal studies. For example, oral supplementation of 2′FL to immunocompromized mice (IL10 null mice) after weaning had a beneficial effect on intestinal inflammation associated with a distinct increase in abundance of *R. gnavus* and greater enrichment of propionate in the caecal content of the mice (Grabinger et al. 2019).

### Secondary metabolites

The importance of *R. gnavus* for host metabolism was first demonstrated using meta-transcriptomics in germ-free mice mono-colonized by different bacterial species (Hoffmann et al. 2016). *R. gnavus* ATCC 29149 induced activation of host pathways involved in tryptophan metabolism and downstream melatonin, nicotin, and serotonin degradation, suggesting a role for these metabolites in the reported association between *R. gnavus* strains and neurological and intestinal disorders (Roager and Licht 2018). Notably, *R. gnavus* ATCC 29149 induced tryptophan hydroxylase 1 and indoleamine 2,3-dioxygenase expression, which was responsible for tryptophan degradation into 5-hydroxytryptophane (5-HTP) (the first step in serotonin and melatonin synthesis) and kynurenine, respectively (Hoffmann et al. 2016). A recent study investigating the role of *R. gnavus* in the gut–brain axis showed, using untargeted metabolomics, that *in vivo* mono-colonization of germ-free mice with *R. gnavus* ATCC 29149 led to an increase in tryptamine (tryptophan metabolism by-product and known neuromodulator of serotonin), indole acetate and host-derived indole acetylglycine (both products of tryptophan metabolism), in the caecal content and serum of gnotobiotic mice compared with germ-free mice (Coletto et al. 2022). Tryptophan decarboxylase, the enzyme decarboxylating tryptophan to tryptamine, has been functionally characterized in *R. gnavus* ATCC 29149 (Williams et al. 2014). To date, *R. gnavus* and *Clostridium sporogenes* are the only bacteria displaying such enzymatic activity in the gut microbiota. Since elevated *R. gnavus* levels have been associated with PD (see section on the 'Association between *R. gnavus* and diseases'), and increased tryptamine levels reported in stress-related disorders and in patients with PD (van Kessel and El Aidy 2019), tryptamine appears to be a *R. gnavus* strain-specific mediator in the gut–brain axis. It is of note that tryptamine also induces mucus release from goblet cells via activation of G-protein-coupled receptor (GPCR) 5-HT4R in a gnotobiotic mouse model mono-colonized with engineered *B. thetaiotaomicron* optimized to produce tryptamine (Bhattarai et al. 2018); this further supports the hypothesis that there is a role for *R. gnavus*-produced tryptamine in the gut–brain axis.

In addition, production of phenethylamine and tryptamine by *R. gnavus*-mediated catabolism of dietary phenylalanine and tryptophan has been shown to directly stimulate serotonin biosynthesis in intestinal enterochromaffin cells of *R. gnavus* ATCC 29149 mono-colonized mice (Zhai et al. 2023). This *R. gnavus*-driven increase in serotonin levels led to IBS-D-like symptoms including elevated GI transit and colonic secretions, which were abrogated upon inhibition of trace amine-associated receptor 1 (Zhai et al. 2023).

Bile acids represent another important regulator of gut microbial composition and are metabolized by gut microbes, such as *R. gnavus*, into secondary bile acids. *R. gnavus* N53 and ATCC 29149 strains produce 7β-hydroxysteroid dehydrogenases (HSDHs) that convert ursodeoxycholic acid (UDCA) into chenodeoxycholic acid (CDCA; 3α, 7α-dihydroxy-5β-cholan-24-oic acid), an important chemopreventive bile acid found in low abundance in humans (Lee et al. 2013). Further, *in silico* analyses of 693 human gut microbial genomes combined with the use of computational tools to analyse phylogenetic trees for protein domains, showed that *R. gnavus* ATCC 29149 is involved in production of secondary bile acids, 3-dehydrocolate and isocholate (3-dehydro-CA and Iso-CA) through the enzymatic action of 3α-HSDH and 3β-HSDH (Heinken et al. 2019); and that its abundance is correlated with production of a specific bile acid metabolite (3-oxoLCA/isoLCA) associated with gut inflammation in IBD patients (Paik et al. 2022).

The demonstrated ability of *R. gnavus* to produce SCFAs, tryptophan and bile acid metabolites is likely to contribute to its role in metabolic disorders and neuro/psychological disorders (see sections on the 'Association between *R. gnavus* and diseases'). Together, these functional investigations *in vitro* and in gnotobiotic mouse models provide important molecular insights underpinning the association between *R. gnavus* strains and intestinal and neurological disorders in humans.

## Conclusions and perspectives

Our understanding of the factors underpinning *R. gnavus* colonization in the gut has been greatly advanced through studies investigating how *R. gnavus* strains use mucin glycans or produce bacteriocins and metabolites *in vitro* and in gnotobiotic mouse models. Despite the variability in human metagenomic studies in terms of sample size, methods of sequencing and analysis, and some confusion over taxonomy, a clear picture is now emerging regarding the potential of *R. gnavus* as a biomarker. Across studies, *R. gnavus* is associated with various intra- and extraintestinal diseases and has a consistent increased representation in IBD and metabolic diseases including obesity, T2D, and NAFLD. These association studies also point towards a role for *R. gnavus* in the gut–brain and gut–liver axis. However, it is still not known whether *R. gnavus* plays an active part in disease development (i.e. a causative role) or benefits from disease-related changes in the microbial ecological niche and physiological status of the host which may favour its expansion in the gut. While some potential mediators of these effects have been characterized in *R. gnavus* strains such as the production of tryptamine, SCFAs, pro-or anti-inflammatory CPS, and secondary bile acids, the causality of *R. gnavus* and associated molecules in human health and disease remains to be uncovered. It is also clear from this review that *R. gnavus* is an integral component of the gut microbiota in infants and adults with some *R. gnavus* strains demonstrating beneficial effects in preclinical models. Since these effects are strain-specific and context-dependent, it is not justified to refer to *R. gnavus* as ‘pro-inflammatory’ or ‘pathogenic’ as sometimes mentioned in metagenomic studies. In addition, despite often being labelled as one of the ‘mucus-degrading bacteria’ in the gut, there is no evidence in the literature to suggest that *R. gnavus* can degrade mucus to the extent that the gut barrier becomes compromised. In contrast some *R. gnavus* strains do promote mucin secretion (as is also the case for *A. muciniphila*), and it is now well-established that the strategy of *R. gnavus* strains to colonize mucus is confined to their ability to use mucin glycan epitopes far away from the epithelium. It is also important to stress that these features are strain specific. We, therefore, suggest referring to these *R. gnavus* strains as mucin glycan foragers rather than mucus degraders. Several questions that need addressing in future studies include: What is the biogeographical distribution of *R. gnavus* strains in the GI tract? Does their capacity to use different carbohydrates enable them to exploit different nutrient niches in the gut? Which *R. gnavus* strains are associated with specific diseases? Do the mediators identified *in vitro* and in mouse models play a causal role in protection from, or development of, diseases? Many of these gaps represent promising opportunities for novel research into how to target *R. gnavus* strains associated with specific diseases through nutritional interventions and, more generally, may advance our broader understanding of the role of mucus-associated microbiota in health and disease.
